# Effect of a Laminarin Rich Macroalgal Extract on the Caecal and Colonic Microbiota in the Post-Weaned Pig

**DOI:** 10.3390/md18030157

**Published:** 2020-03-11

**Authors:** Stafford Vigors, John V O’Doherty, Ruth Rattigan, Mary J McDonnell, Gaurav Rajauria, Torres Sweeney

**Affiliations:** 1 School of Agriculture and Food Science, University College Dublin, Belfield, Dublin 4, D04 V1W8, Ireland; staffordvigors1@ucd.ie (S.V.); ruth.rattigan@ucdconnect.ie (R.R.); marymcdonnell06@gmail.com (M.J.M.); gaurav.rajauria@ucd.ie (G.R.); 2 School of Veterinary Medicine, University College Dublin, Belfield, Dublin 4, D04 V1W8 Ireland; torres.sweeney@ucd.ie

**Keywords:** gut microbiome, weaning, pigs, seaweed, laminarin

## Abstract

Dietary supplementation with 300 ppm of a laminarin rich macroalgal extract reduces post-weaning intestinal dysfunction in pigs. A comprehensive analysis of the impact of laminarin on the intestinal microbiome during this period is essential to inform on the mode of action of this bioactivity. The objective of this study was to evaluate the effects of supplementing the diet of newly weaned pigs with 300 ppm of a laminarin rich extract, on animal performance, volatile fatty acids, and the intestinal microbiota using 16S rRNA gene sequencing. Pigs fed the laminarin-supplemented diet had higher average daily feed intake, growth rate, and body weight compared to pigs fed the control diet (*p* < 0.05). Pigs fed the laminarin-supplemented diet had reduced abundance of OTUs assigned to *Enterobacteriaceae* and increased abundance of OTUs assigned to the genus *Prevotella* (*p* < 0.05) compared to pigs fed the control diet. *Enterobacteriaceae* had negative relationships (*p* < 0.05) with average daily feed intake (ADFI), average daily gain (ADG), and butyric acid concentrations. In contrast, *Prevotellaceae* were positively correlated (*p* < 0.05) with ADFI, ADG, total VFA, acetic, propionic, butyric acids, and negatively correlated with isovaleric acid. Hence supplementation with a laminarin enriched extract potentially improves performance during the post-weaning period by promoting the proliferation of bacterial taxa such as *Prevotella* that favourably enhance nutrient digestion while reducing the load of potentially pathogenic bacterial taxa including *Enterobacteriaceae*.

## 1. Introduction

Weaning is characterised by a reduction in feed intake resulting in villus atrophy, compromised gut barrier function and microbial instability [1]. This reduction in feed intake also leads to reduced substrate availability for microbial fermentation, reducing bacterial diversity and provides an opportunity for the proliferation of pathogenic bacteria, such as enterotoxigenic *Escherichia coli* (ETEC), leading to post-weaning diarrhoea (PWD) [2]. The pathogenicity of bacterial strains such as ETEC is enhanced due to multifactorial stressors such as removal from the sow, dietary changes, adapting to a new environment, mixing of pigs from different farms and histological changes in the small intestine, which may negatively affect the response of the immune system and lead to intestinal dysbiosis in pigs [3]. In-feed antibiotics or high concentrations of zinc oxide are widely administered to control post-weaning diarrhoea and to improve performance in pigs during the post-weaning period [4]. However, the use of antibiotics as growth promoters has been banned in the EU since 2006 (Regulation (EC) No. 1831/2003 and 1334/2003) and the EU will phase out zinc oxide at medicinal levels and prohibit the use of medicated feed for prophylaxis purposes by 2022. Hence, the exploration of alternatives which can be administered over the vulnerable weaning period is a priority in pig production.

The development of nutritional strategies that promote beneficial microorganisms and thus maintain gastrointestinal (GIT) homeostasis is one approach to improve gut health [5,6]. Intestinal microorganisms are important in terms of regulating digestion and improving host performance through the fermentation of non-digestible polysaccharides, resulting in the production of beneficial volatile fatty acids (VFA), particularly butyrate [5,7]. Furthermore, the colonisation of the intestinal mucosa and lumen by commensal bacteria reduces the accessibility of pathogens to both nutrients and attachment sites [8,9]. Marine macroalgal derivatives have been considered as potential bioactive compounds in swine nutrition and in particular as a prebiotic that can favourably support the intestinal microbiome [4,10,11]. Brown algae are a rich source of laminarin and fucoidan which are marine derived polysaccharides that have a range of bioactive properties both in vitro and in vivo [1,11,12]. The addition of laminarin to the diet improves nutrient digestibility through the upregulation of nutrient transporter gene expression, enhanced villus structure and higher production of VFAs [10,13,14,15]. β-glucans such as laminarin, have the capacity to agglutinate bacterial species, inhibiting attachment and the colonisation of mucosal surfaces by pathogenic bacteria such as *Escherichia coli*, but these analyses have been limited to the evaluation of specific bacterial taxa in pigs [1,14,16,17,18]. A more comprehensive understanding of the impact of laminarin on the microbiome of pigs is attainable using genomic approaches, such as employing 16s rRNA sequencing, as this technique can measure the relative proportions of a wide range of bacterial taxa. This will ultimately provide an understanding of both the anti-microbial and prebiotic potential of laminarin. Using high throughput 16s rRNA sequencing, polysaccharides from *Enteromorpha clathrata* (ECP) altered the intestinal microbiome in mice with increased abundances of *Bifidobacterium* spp. and *Akkermansia muciniphila* in females and Lactobacillus spp. in males [19]. Hence, the objective of this study was to evaluate the effects of supplementing newly weaned piglets with 300 ppm of a laminarin rich extract on the caecal and colonic microbiota through 16s rRNA sequencing. It is hypothesized that pigs supplemented with a laminarin rich extract will display a more favourable gut microbiota evidenced by reductions in pathogenic bacteria and increases in bacterial taxa associated with improved health and nutrient breakdown.

## 2. Results

### 2.1. Animal Performance and Faecal Scores 

During the 14-day experimental period, pigs fed the laminarin supplemented diet had higher ADFI (0.31 vs 0.38 kg/day, SEM 0.01, *p* < 0.01), ADG (0.16 vs 0.22 kg/day, SEM 0.02, *p* < 0.05) and final body weight (9.78 vs 10.23 kg, SEM 0.20, *p* < 0.01) compared to pigs fed the control diet. Faecal scores were similar between the two groups (2.8 vs 2.6) throughout the experiment (*p* > 0.05), with animals from both the basal and laminarin supplemented groups having faecal scores in the healthy range.

### 2.2. Volatile Fatty Acid Analysis 

The effects of laminarin supplementation on caecal and colonic VFA are presented in Table 1. No differences were observed in the caecum. Pigs fed a diet supplemented with laminarin had higher total colonic VFA (*p* < 0.01) and butyric acid concentrations (*p* < 0.05) compared to pigs fed the control diet. 

### 2.3. Large Intestinal Microbiota 

The effect of supplementation with a 300 ppm laminarin rich macroalgal extract on the large intestinal microbiota was determined using next-generation sequencing of the 16S rRNA gene, using the Illumina MiSeq platform as detailed in the materials and methods. Bioinformatic analysis, as described in the materials and methods, allowed for the identification of 957 OTUs. The full 16S rRNA microbial analysis data for both the caecum and colon are presented in the supplementary materials (Tables S1–S5 and Figures S1–S3).

#### 2.3.1. Bacterial Richness and Diversity Analysis 

Alpha diversity in the caecum and colon was similar between the laminarin supplemented and control pigs (Table 2; *p* > 0.10). In relation to beta diversity, the pigs did not cluster based on the main parameters in this study, i.e., neither diet nor region of the gastrointestinal tract (Figure S1). As beta diversity is a measure of between animal variation, it suggests that there is large variation between individuals in this study. 

#### 2.3.2. Differential Abundance Analysis of Bacterial Taxa in Caecal Digesta and Their Correlations with Performance Traits

**Figure 1:**
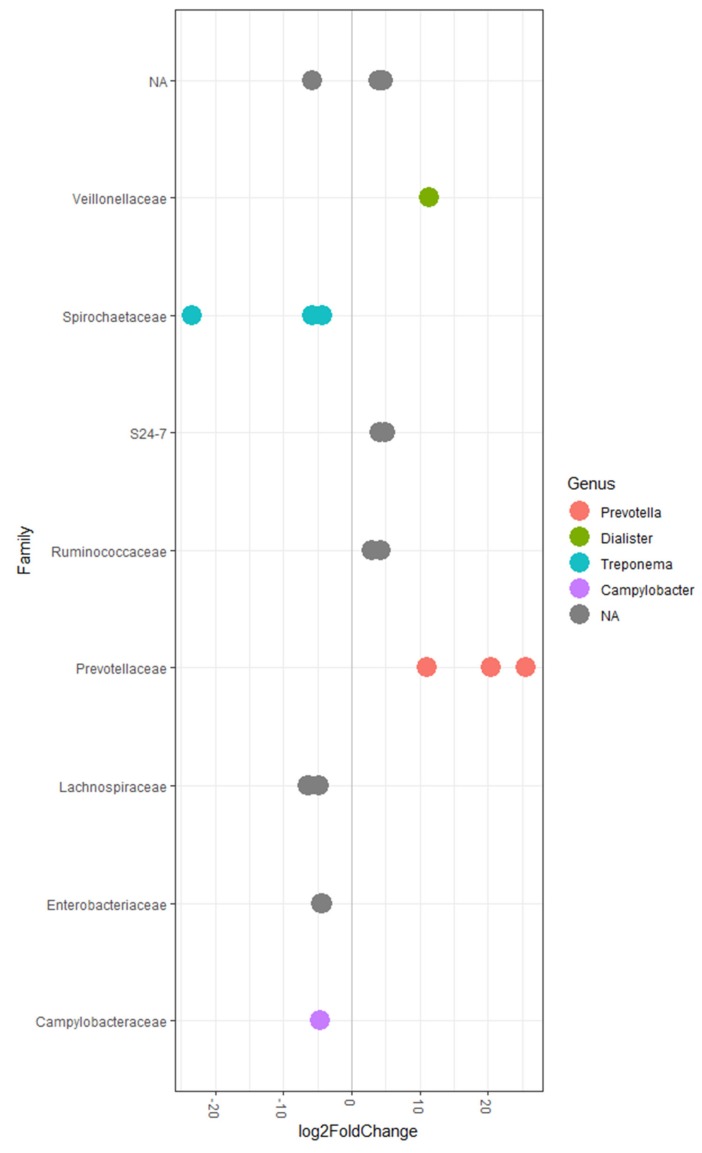
Identification of differentially abundant OTUs in the caecum of pigs fed a basal diet supplemented with a laminarin enriched macroalgal extract compared to the basal control diet. The *y*-axis displays the family that the OTU was assigned to, while the colours illustrate the genus the OTU was assigned to if it was possible to assign. A negative log2FoldChange indicates a reduction, while a positive log2FoldChange indicates an increase in abundance.

The 20 OTUs that are significantly different between the control and laminarin supplemented groups are presented in Figure 1 and Table S1 with the top 20 most abundant OTUs presented in Figure S2. Analysis of differential abundance of bacterial taxa at phylum, family, genus, and species level are presented as percentages in Table S2. The correlations between taxonomic families and performance traits are presented in Table 3. 

Within the phylum Bacteroidetes, the abundance of the genus *Prevotella* was increased in pigs fed the laminarin supplemented diet compared to pigs on the control diet (*p* < 0.10; Table S2). Three OTUs (36031, 299882, 4397200) assigned to the genus *Prevotella* had greater abundance (*p* < 0.05) in the laminarin supplemented pigs compared to the control pigs. *Prevotellaceae* was positively correlated with ADFI (r = 0.56; *p* < 0.05), ADG (r = 0.59; *p* < 0.05), total VFA (r = 0.76; *p* < 0.001), acetic (r = 0.68; *p* < 0.01), propionic ( r= 0.64; *p* < 0.01), butyric acid (r = 0.56; *p* < 0.05), and negatively correlated with isovaleric acid (r = 0.75; *p* < 0.001). Two OTUs (New.ReferenceOTU3159 and 1652947) assigned to the family *S24-7* had greater abundance in pigs fed the laminarin supplemented diet. While not differentially abundant between the control and laminarin supplemented pigs, two families within the phylum Bacteroidetes were negatively correlated with measures of performance: the family *Bacteroidaceae* was negatively correlated with ADFI (r = −0.73; *p* < 0.01), ADG (r = −0.65; *p* < 0.05), and butyric acid concentrations (r = −0.65; *p* < 0.05); and the family *BS11* was negatively correlated with ADFI (r = −0.74; *p* < 0.01), ADG ( r= −0.62; *p* < 0.01), and butyric acid concentrations (r = −0.72; *p* < 0.01). Similarly, *Porphyromonadaceae* was negatively correlated with ADFI (r = −0.75; *p* < 0.01), ADG ( r= −0.60; *p* < 0.01) and butyric acid concentrations (r = −0.68; *p* < 0.01). 

Within the phylum Firmicutes, nine differentially abundant OTUs were identified and assigned to the families *Ruminococcaceae*, *Veillonellaceae*, and *Lachnospiraceae* (Figure 1; Table S1). 

Within the phylum Proteobacteria, the abundance of the family *Enterobacteriaceae* was reduced in the pigs fed the laminarin supplemented diet compared to pigs on the control diet (*p* < 0.05; Table S2). It must be noted that the elevated abundance in the control animals was predominately based on increased abundance in three of the nine control pigs (Figure S3). Two OTUs (1111294, 797229) assigned to *Enterobacteriaceae* (*p* < 0.05) were reduced in pigs fed the laminarin supplemented diet compared to pigs on the control diet. *Enterobacteriaceae* were negatively correlated with ADG (r = −0.63; *p* < 0.01), butyric acid (r = −0.63; *p* < 0.01), and positively correlated with iso-valeric acid (r = 0.56; *p* < 0.05). An additional OTU (9302158) was assigned to the genus Campylobacter and was reduced in the laminarin supplemented pigs compared to controls. While *Helicobacteraceae* was not different between the control and supplemented groups, it was negatively correlated with ADFI (r = −0.75; *p* < 0.01), ADG (r = −0.63; *p* < 0.01), and butyric acid concentrations (r = −0.67; *p* < 0.01). 

Within the phylum *Spirochaetes*, three OTUs (New.ReferenceOTU968, 924224, New.ReferenceOTU282) assigned to the genus *Treponema* had lower abundance (*p* < 0.05) in the laminarin supplemented pigs compared to the control pigs.

#### 2.3.3. Differential Abundance Analysis of Bacterial Taxa in Colonic Digesta and Their Correlations with Performance Traits

The three OTUs that are significantly different in the colon between the control and laminarin supplemented groups are presented in Table S3. Analysis of differential abundance of bacterial taxa at phylum, family, genus, and species level are presented as percentages in Table S4. The correlations between taxonomic families and performance traits are presented in Table 4. 

Within the phylum Bacteroidetes, one OTU (New Reference OTU 1384) assigned to the species *Prevotella copri* had greater abundance (*p* < 0.05) in the laminarin supplemented pigs compared to the control pigs. The abundance of the family *P-2534-18B5* was decreased in pigs fed the laminarin supplemented diet compared to pigs on the control diet (*p* < 0.05). *Prevotellaceae* was positively correlated with ADFI (r = 0.53; *p* < 0.05), ADG (r = 0.59; *p* < 0.01), total VFA (r = 0.62; *p* < 0.01), acetic (r = 0.49; *p* < 0.05), propionic (r = 0.69; *p* < 0.001), butyric acid (r = 0.63; *p* < 0.01), and valeric acid (r = 0.75; *p* < 0.001). In addition, *Porphyromonadaceae* was negatively correlated with ADFI (r = −0.77; *p* < 0.001) and butyric acid (r = −0.61; *p* < 0.01). *Bacteroidaceae* was negatively correlated with ADFI (r = −0.75; *p* < 0.001). 

Within the phylum *Spirochaetes*, one OTU (New Reference OTU 968) assigned to the genus *Treponema* had lower abundance in pigs fed the laminarin supplemented diet compared to pigs fed the control diet (*p* < 0.05).

## 3. Discussion

The current study hypothesized that pigs fed a diet supplemented with 300 ppm of a laminarin rich extract will result in increases in taxa that support improved health and nutrient breakdown/absorption and reductions in potentially pathogenic bacteria. The results from this study supported this hypothesis as the laminarin supplemented pigs had reductions in important pathogenic bacterial taxa, such as the family *Enterobacteriaceae.* This finding coincided with evidence for higher abundance of the beneficial *Prevotella*, which was positively associated with average daily feed intake, average daily gain, and volatile fatty acid production, in particular butyric acid. These associated correlations suggest that this microbial profile underlies the improved performance of the laminarin supplemented pigs. 

The addition of laminarin to the diet of pigs in the post weaning period had beneficial effects on animal performance and had an influence the production of volatile fatty acids. These beneficial effects have been discussed in detail in the work of Rattigan et al. [20] and therefore, will not be discussed extensively in this paper where the focus will be on the effects on the bacterial population. Consistent with previous studies, Proteobacteria, Bacteroidetes, and Firmicutes were the three most dominant phyla in the weaned pigs, with Bacteroidetes being most abundant [21,22,23]. Within the phylum Proteobacteria, the control pigs had a greater abundance of the family *Enterobacteriaceae* compared to the laminarin supplemented pigs (Figure 1). Members of the phylum Proteobacteria are early transient colonisers in the post-weaning period and are later replaced by more stable bacterial populations [24]. The *Enterobacteriaceae* family consists of numerous pathogenic bacteria including *Escherichia coli (E. coli)*, *Salmonella, Klebsiella*, and *Shigella*. Previously, *Enterobacteriaceae* has been suggested as an indicator of pathogenic bacteria as higher coliform counts were observed in the intestine of scouring pigs and the density of coliforms is used as an indicator of *E. coli* and *Salmonella* [25,26]. *E. coli* proliferation is one of the main causes of post-weaning diarrhoea in pigs [1,2]. Previously, laminarin supported growth during an ETEC challenge with supplemented pigs having improved growth rates and reduced diarrhea compared with pigs fed the control unsupplemented diet [18]. Similarly, the supplementation of laminarin to pigs suppressed *Enterobacteriaceae* populations and lowered faecal *E. coli* numbers [10,16,27]. The reduction in *Enterobacteriaceae* suggests an overall improvement in gut health in the laminarin supplemented pigs (Figure 1). It must be noted that within the control pigs, *Enterobacteriaceae* abundance was elevated in only three pigs. This is interesting as all of the pigs came from the same litters and the same farrowing house. There is evidence to suggest that there is a genetic component to susceptibility to *E. coli* infection in pigs with only a proportion of the population naturally susceptible [28]. At a family level, it is clear from the correlations that members of the phylum Proteobacteria (*Enterobacteriaceae*, *Helicobacteraceae),* phylum Bacteroidetes *(Bacteroidaceae*, *Porphyromonadaceae*, and *BS-11)* and phylum Fusobacterium (*Fusobacteriaceae)* are negatively associated with animal growth and the production of VFA’s (Table 3).

Within the phylum Bacteroidetes, *Prevotella* was the most abundant genus in both the caecum and colon with a greater abundance observed in the laminarin supplemented pigs compared to controls and *Prevotellaceae* was positively correlated with measures of animal performance (Figure 1 & Table 3 and Table 4). *Prevotella* have the capacity to degrade dietary xylan from cereal grains [29]. Similarly, *Prevotella bryantii* breaks down soluble xylans by producing xylanases, mannanases, and β-glucanase in non-ruminants suggesting higher capacity to digest fibre [30]. Previous research in mice established that supplementation with *Enteromorpha clathrata* increased the abundance of *Prevotella* spp. [19]. In our study, *Prevotellaceae* was significantly correlated with acetic, butyric, propionate and total VFA production suggesting that the higher *Prevotella* is associated with higher fibre breakdown and VFA production (Table 3). This is in agreement to previous studies where laminarin supplementation led to higher total tract nutrient digestibility [15]. *Prevotellaceae* is also strongly correlated with ADG in the current study. This strong correlation with animal performance suggests that *Prevotella* is a key factor impacting the improved performance of the laminarin supplemented pigs. Previously, as pigs transition from pre- to post-weaning there is a shift from high numbers of *Enterobacteriaceae* to high numbers of *Prevotellaceae* [24]. One possible explanation for the results in this study is that laminarin supplementation accelerates the microbial transition to a settled post-weaning microbiome. This post-weaning microbiome may therefore, have a greater capacity to breakdown the diet, driving the improved performance in the laminarin supplemented pigs. Zhang et al. [31] identified a co-exclusion relationship between *Enterobacteriaceae* and *Prevotellaceae*. These authors highlighted the greater capacity of members of the *Prevotellaceae* family to digest complex carbohydrates into VFAs which can directly reduce the colonization of *Enterobacteriaceae* by lowering intestinal pH and reducing inflammation. Conversely, the laminarin supplemented pigs have higher ADFI which in turn may be influencing microbial succession as the introduction of solid feed is known to influence microbial maturation [32]. Based on the evidence from this study it seems that laminarin supplementation has a beneficial effect on animal performance and the intestinal microbial population. However, while the extraction procedure was designed to maximise the extraction of laminarin, other compounds—such as fucoidan, phlorotannins, mannitol and alginates—may be having an impact. For example fucoidan has been shown to alter gut microbiota for health promotion and the treatment of intestinal dysbiosis in mice [12]. Further research will need to be conducted to assess fully purified extracts in isolation from other compounds.

## 4. Materials and Methods 

All experimental procedures described in this work were approved under the University College Dublin Animal Research Ethics Committee (AREC-17-19-O’Doherty) and were conducted in accordance with Irish legislation (SI no. 543/2012) and the EU directive 2010/63/EU for animal experimentation. 

### 4.1. Experimental Design, Animal Management, and Diets 

At 28 days of age, newly weaned pigs (n = 54) [progeny of Landrace boars x (Large White x Landrace) sows] were sourced from sows from the same farrowing house. Piglets were weighed and blocked across houses on the basis of initial live weight (8.4 kg, sd- 1.1 kg) litter of origin and sex and assigned to one of two groups: (1) basal diet; and (2) basal diet + 300 ppm of a laminarin rich extract. This inclusion level was determined based on previous work by Rattigan et al. [20], where supplementation at 300 ppm improved animal performance and digestive health. The basal diet contained 15.3 MJ/kg digestible energy, 190 g/kg crude protein and 13.5 g/kg total lysine. The basal diet was comprised of wheat (34%), extruded full fat soya (17%), flaked wheat (13%) HIPRO soya (soybean meal (48% crude protein) (10.5%), flaked maize (7%) whey powder (5%), provisoy (soya protein concentrate) (6.5%), soya oil (3%) with the remainder compromised of mineral and vitamin supplements (Table 5). All amino acid requirements were calculated relative to lysine. The laminarin enriched extract was sourced from BioAtlantis Ltd (Clash Industrial Estate, Tralee, Co. Kerry, Ireland). The laminarin rich extract was obtained from Laminaria spp using hydrothermal assisted extraction by applying optimised extraction conditions as described in our previous publication [33]. The crude extract was partially purified in order to enhance polysaccharide content and to remove or reduce proteins, polyphenols, and the excess amount of mannitol and alginate, by mixing the crude extract with pure ethanol (to remove polyphenols) followed by water (to remove protein) and calcium chloride (to remove alginates). The extract contained 653.2 g of laminarin per kg DM, 190 g/kg fucoidan per kg DM, 5 g/kg phlorotannin per kg DM, 51 g/kg mannitol per kg DM, 40 g/kg alginates per kg DM and 3.18 g/kg ash per kg DM.

The pigs were penned in groups of three and housed on fully slatted floors (1.68 × 1.22 m). The pigs were individually weighed at the beginning of the experiment (day 0 = day of weaning) and subsequently on days 7 and 14. The ambient temperature within the house was thermostatically controlled at 30 °C and humidity was maintained at 65%. The feed was in meal form and was available ad libitum up to the final weighing. Faecal scores of the pigs were taken daily where 1 = hard firm faeces; 2 = slightly soft faeces; 3 = soft, partially formed faeces; 4 = loose, semi-liquid faeces; 5 = watery, mucous-like faeces. On day 15, nine pigs per treatment (one from each pen) were humanely euthanized. The entire intestinal tract was removed immediately. Digesta from the caecum and colon was collected and stored in sterile containers (Sarstedt, Wexford, Ireland) and immediately frozen (–20 °C) for subsequent 16s rRNA sequencing and VFA analyses.

### 4.2. Feed Analysis

The feed samples were milled through a 1 mm screen (Christy and Norris hammer mill, Ipswich, UK). The dry matter (DM) of the feed was determined after drying overnight at 104 °C. Crude ash content was determined after the ignition of a known weight of concentrate in a muffle furnace (Nabertherm, Bremen, Germany) at 550 °C for 6 h. The crude protein (CP) content was determined as Kjeldahl N × 6.25 using the LECO FP 528 instrument. The neutral detergent fibre (NDF) content was determined according to Van Soest et al. [35]. 

### 4.3. Volatile Fatty Acid Analysis 

The VFA concentrations in the digesta were determined using gas liquid chromatography (GLC) according to the method described by Clarke et al. [36]. A 1-g sample was diluted with distilled water (2.5 × weight of sample) and centrifuged at 1400 g for 10 min (Sorvall GLC-2B laboratory centrifuge, DuPont, Wilmington, DE, USA). One mL of the subsequent supernatant and 1 mL of internal standard (0.05% 3-methyl-n-valeric acid in 0.15 M oxalic acid dihydrate) were mixed with 3 mL of distilled water. The reaction mixture was centrifuged at 500 g for 10 min, and the supernatant was filtered through 0.45 PTFE syringe filter into a chromatographic sample vial. An injection volume of 1 μL was injected into a Varian 3800 GC equipped with a EC™ 1000 Grace column (15 m × 0.53 mm I.D) with 1.20 μm film thickness. The temperature programme set was 75–95 °C increasing by 3 °C/min, 95–200 increasing by 20 °C per minute, which was held for 0.50 min. The detector and injector temperature was 280 °C and 240 °C, respectively, while the total analysis time was 12.42 min.

### 4.4. Microbiological Analyses

#### 4.4.1. Microbial DNA Extraction 

Microbial genomic DNA was extracted from the caecal and colonic digesta samples using a QIAamp DNA stool kit (Qiagen, West Sussex, UK) in accordance with the manufacturer’s instructions. The quantity and quality of DNA were assessed using a Nanodrop spectrophotometer (Thermo Scientific, Wilmington, DE, USA).

#### 4.4.2. Illumina Sequencing

High-throughput sequencing of the V3-V4 hypervariable region of the bacterial 16S rRNA gene was performed on an Illumina MiSeq platform according to their standard protocols (Eurofins, Wolverhampton, UK). Briefly, the V3–V4 region was PCR-amplified using universal primers containing adapter overhang nucleotide sequences for forward and reverse index primers. Amplicons were purified using AMPure XP beads (Beckman Coulter, Indianapolis, IN, USA) and set up for the index PCR with Nextera XT index primers (Illumina, San Diego, CA, United States). The indexed samples were purified using AMPure XP beads, quantified using a fragment analyzer (Agilent, Santa Clara, CA, USA), and equal quantities from each sample were pooled. The resulting pooled library was quantified using the Bioanalyzer 7500 DNA kit (Agilent) and sequenced using the V3–V4 chemistry (2 × 300 bp paired-end reads). 

### 4.5. Bioinformatic and Statistical Analyses

The resulting sequences were analysed using the open source software package Quantitative Insights into Microbial Ecology (Qiime) [37]. Initially, sequencing primers were removed using the cutadapt function of Qiime. Paired-end reads were then joined with the multiple join paired-end reads function within Qiime using the default parameters. Using the split libraries function, the raw reads were initially demultiplexed, and reads were quality filtered using default QIIME parameters and sequences that contained ambiguous characters, non-exact barcode matches, sequence length < 225 nucleotides and having a read-quality score of < 27 were removed. OTUs were picked at 97% sequence similarity using the uclust function within Qiime. Singletons were removed, as only OTUs that were present at the level of at least two reads in more than one sample were retained. The resulting OTU representative sequences were assigned to different taxonomic levels (from phylum to species) using the GreenGenes database. Chimeras were identified and removed with the use of ChimeraSlayer [37,38]. The normalized OTU table combined with the phenotype metadata and phylogenetic tree comprised the data matrix. This matrix was then input into the phyloseq package within the R (http://www.r-project.org; version 3.5.0). The dynamics of richness and diversity in the piglet’s microbiota were computed with the observed, the Simpson and the Shannon indices. The Simpson and Shannon indices of diversity account for both richness and evenness parameters. To estimate beta diversity measurements, which are a measure of separation of the phylogenetic structure of the OTU in one sample compared with all other samples, the data was normalised to make taxonomic feature counts comparable across samples. Several distance metrics were considered, in order to calculate the distance matrix of the different multidimensional reduction methods. These included weighted/unweighted UniFrac distance and non-phylogenetic distance metrics (i.e., Bray-Curtis, Jensen-Shannon divergence and Euclidian) using phyloseq in R [39,40]. Taxonomy and diversity plots were produced using graphics tailored for phylogenetic analysis using the R package ggplot2 [41]. Differential abundance testing was performed using the phyloseq to deseq2 function within R [40,42]. Results are presented using Benjamini–Hochberg (BH) adjusted *p*-values. 

All other data were initially checked for normality using the univariate procedure of Statistical Analysis Software (SAS) 9.4 (SAS Institute, Cary, NC, USA). The performance data and FS data were analysed using repeated measures within the mixed procedure of SAS, and the model included fixed effects of treatment, time and their associated interactions. The initial weight was used as a covariate for the performance data. The model assessed the effect of treatment, with the pig being the experimental unit. The probability level that denoted significance was *p* < 0.05, while *p*-values between 0.05 and 0.1 are considered numerical tendencies. Data are presented as least-square means with their standard errors of the mean.

## 5. Conclusions

The feeding of a 300 ppm laminarin rich extract during the post-weaning period has a positive influence on intestinal health and is potentially a driver of improved animal performance. Pigs fed a diet supplemented with 300 ppm of a laminarin rich extract had increases in taxa such as *Prevotella*, which is associated with higher nutrient breakdown and utilisation as well as a lower abundance of *Enterobacteriaceae*. Hence the intestinal microbiome of laminarin supplemented pigs is better suited to the digestion of a classical cereal-based post-weaning diet resulting in improved animal performance during this critical period. This suggests possible use of laminarin in minimising intestinal disorders in both animals and humans.

## Figures and Tables

**Table 1:** Effects of laminarin supplementation on concentrations (mmol/g digesta) of caecal and colonic volatile fatty acids (VFA) (Least-square mean values with their standard errors).

	Control	Laminarin	SEM	*p* value
**Caecum**				
Total VFA^1^	127.55	137.94	6.49	0.274
Acetic acid	92.57	98.85	4.79	0.368
Propionic acid	19.85	21.75	1.55	0.401
Butyric acid	12.76	15.15	1.29	0.211
Isobutyric acid	0.63	0.45	0.11	0.313
Isovaleric acid	0.45	0.44	0.06	0.879
Valeric acid	1.27	1.28	0.18	0.980
Ace:Prop ratio	4.69	4.76	0.29	0.870
**Colon**				
Total VFA	130.62	157.83	7.37	0.027
Acetic acid	95.36	107.12	6.03	0.034
Propionic acid	19.04	22.26	1.91	0.094
Butyric acid	11.77	17.96	1.78	0.050
Isobutyric acid	1.03	1.23	0.22	0.536
Isovaleric acid	0.89	1.2	0.136	0.121
Valeric acid	1.27	1.28	0.18	0.101
Ace:Prop ratio	5.11	5.01	0.18	0.822

^1^ VFA = volatile fatty acid; Ace:Prop = acetic:propionic acid ratio.

**Table 2:** Effects of laminarin supplementation on measures of alpha diversity in caecal and colonic digesta

	Control	Laminarin	SEM	*p* value
**Caecum**				
Observed	646.11	648.38	17.15	0.951
Chao1	697.07	707.97	16.70	0.763
ACE	686.77	695.85	15.82	0.791
Shannon	3.92	3.93	0.11	0.971
Simpson	0.94	0.92	0.01	0.459
InvSimpson	19.12	19.89	2.60	0.892
Fisher	86.93	87.04	2.66	0.985
**Colon**				
Observed	689.50	705.00	16.16	0.657
Chao1	739.06	748.56	13.40	0.743
ACE	725.70	735.86	14.22	0.741
Shannon	4.43	4.47	0.11	0.886
Simpson	0.96	0.95	0.01	0.615
InvSimpson	39.29	32.07	4.93	0.496
Fisher	94.48	96.72	2.49	0.679

**Table 3:** Correlations between bacterial taxa at family level and performance and volatile fatty acids in the caecum

Taxa	Trait	Correlation	AdjPvalue
*Prevotellaceae*	Acetic	0.68	0.01
*Porphyromonadaceae*	ADFI	-0.76	0.00
*Helicobacteraceae*	ADFI	-0.75	0.00
*BS11*	ADFI	-0.75	0.00
*Bacteroidaceae*	ADFI	-0.74	0.00
*Fusobacteriaceae*	ADFI	-0.73	0.01
*Prevotellaceae*	ADFI	0.53	0.03
*Helicobacteraceae*	ADG	-0.63	0.01
*BS11*	ADG	-0.63	0.02
*Porphyromonadaceae*	ADG	-0.61	0.02
*Enterobacteriaceae*	ADG	-0.60	0.04
*Bacteroidaceae*	ADG	-0.58	0.04
*Fusobacteriaceae*	ADG	-0.57	0.04
*Prevotellaceae*	ADG	0.59	0.02
*BS11*	Buty	-0.72	0.00
*Porphyromonadaceae*	Buty	-0.68	0.01
*Helicobacteraceae*	Buty	-0.68	0.01
*Bacteroidaceae*	Buty	-0.66	0.01
*Fusobacteriaceae*	Buty	-0.65	0.01
*Enterobacteriaceae*	Buty	-0.63	0.04
*Prevotellaceae*	Buty	0.56	0.02
*Prevotellaceae*	IsoVal	-0.56	0.02
*Enterobacteriaceae*	IsoVal	0.56	0.05
*p.2534.18B5*	IsoVal	0.65	0.03
*Mogibacteriaceae.*	IsoVal	0.75	0.00
*Prevotellaceae*	Prop	0.64	0.01
*Prevotellaceae*	Total VFA	0.76	0.00

**Table 4:** Correlations between bacterial taxa at family level and performance and volatile fatty acids in the colon

Taxa	Trait	Correlation	AdjPvalue
*Prevotellaceae*	Acetic	0.49	0.05
*Porphyromonadaceae*	ADFI	-0.77	0.00
*Bacteroidaceae*	ADFI	-0.75	0.00
*Fusobacteriaceae*	ADFI	-0.73	0.01
*Helicobacteraceae*	ADFI	-0.71	0.01
*Prevotellaceae*	ADFI	0.53	0.04
*Prevotellaceae*	ADG	0.59	0.02
*Prevotellaceae*	Buty	0.63	0.01
*Porphyromonadaceae*	Buty	-0.61	0.03
*Prevotellaceae*	Prop	0.69	0.01
*Prevotellaceae*	Total VFA	0.63	0.01
*Prevotellaceae*	Valer	0.75	0.00

**Table 5:** Ingredient and chemical composition of basal diet*

Ingredient (g/kg)	
Wheat	340.0
Full fat soya	170.0
Flaked wheat	130.0
Soya bean meal	105.0
Flaked maize	70.0
Whey powder	50.0
Soya oil	65.0
Vitamins and minerals	2.5
Sodium bicarbonate	2.0
Mono calcium phosphate	4.0
Calcium carbonate (Limestone)	6.0
Salt	2.0
Lysine HCL	4.0
DL-methionine	1.5
L-threonine	1.5
**Chemical analysis**	
DM	866.1
Crude protein (N × 6.25)	190
Digestible energy (MJ/kg) †	14.95
Ash	48.4
Neutral detergent fibre	114.00
Lysine †	13.5
Methionine and cysteine †	7.4
Threonine †	7.9
Tryptophan †	2.6
Calcium †	7.2
Phosphorous †	6.0

*Treatments were as follows: (1) basal diet; (2) basal diet + 300 parts per million (ppm) of a laminarin rich extract; † Values calculated based on tabulated nutritional composition [34]. The basal diet was formulated to provide: Cu, 100; Fe, 140; Mn, 47; Zn, 120; I, 0.6; Se, 0.3; retinol, 1.8; cholecalciferol, 0.025; α-tocopherol, 67; phytylmenaquinone, 4; cyanocobalamin, 0.01; riboflavin, 2; nicotinic acid, 12; pantothenic acid, 10; choline chloride, 250; thiamine, 2; pyridoxine, 0.015 (mg/kg diet). Celite was also included at 300 mg/kg.

## Supplementary Materials

The following are available online at https://www.mdpi.com/1660-3397/18/3/157/s1, Table S1: This file contains the significantly differentially abundant OTUs identified in the caecal digesta. Table S2: This file contains the results of differential abundance analysis for all taxa in the caecum presented as percentages Table S3: This file contains the significantly differentially abundant OTUs identified in the colonic digesta. Table S4: This file contains the results of differential abundance analysis for all taxa in the colon presented as percentages. Table S5: This file contains the results of the PICRUSt analysis in the caecal digesta. Figure S1: Beta diversity analysis using multiple dimensional scaling and bray-curtis distance matrix. Figure S2: Heatmap showing the top 20 most abundant OTUs. Figure S3: Taxonimic distribution of individual samples at family level.
