# Supplementary material for: Effect of a Laminarin Rich Macroalgal Extract on the Caecal and Colonic Microbiota in the Post-Weaned Pig
Source: Mar Drugs. 2020 Mar 11;18(3):157. doi: 10.3390/md18030157 (PMC7143854; doi:10.3390/md18030157)
Supplement: Supplementary file 1 [file marinedrugs-18-00157-s001.pdf]

Table S1. Differential abundance analysis of the 20 significant OTUS from the caecum of pigs fed either a control or laminarin supplemented diet

OTU	baseMean	log2FoldChange	lfcSE	pvalue	padj	Phylum	Family	Genus	Species
New.ReferenceOTU3159	19.28	4.93	1.40	0.000	0.039	Bacteroidetes	S24-7	NA	NA
1652947	8.88	4.17	1.26	0.001	0.049	Bacteroidetes	S24-7	NA	NA
36031	156.60	25.47	2.96	0.000	0.000	Bacteroidetes	Prevotellaceae	Prevotella	NA
299882	17.09	20.42	2.97	0.000	0.000	Bacteroidetes	Prevotellaceae	Prevotella	NA
4397200	128.78	11.04	2.96	0.000	0.026	Bacteroidetes	Prevotellaceae	Prevotella	NA
264552	127.62	11.41	2.96	0.000	0.019	Firmicutes	Veillonellaceae	Dialister	NA
640999	17.49	4.56	1.15	0.000	0.015	Firmicutes	NA	NA	NA
355697	57.58	-6.41	1.74	0.000	0.026	Firmicutes	Lachnospiraceae	NA	NA
47477	30.94	-6.23	1.75	0.000	0.037	Firmicutes	Lachnospiraceae	NA	NA
New.ReferenceOTU1338	55.19	-4.74	1.45	0.001	0.049	Firmicutes	Lachnospiraceae	NA	NA
New.ReferenceOTU375	19.51	-5.71	1.76	0.001	0.049	Firmicutes	NA	NA	NA
4322752	4.24	3.91	1.20	0.001	0.049	Firmicutes	NA	NA	NA
392918	60.90	4.25	1.29	0.001	0.049	Firmicutes	Ruminococcaceae	NA	NA
362078	128.72	2.99	0.93	0.001	0.049	Firmicutes	Ruminococcaceae	NA	NA
302158	8.19	-4.63	1.41	0.001	0.049	Proteobacteria	Campylobacteraceae	Campylobacter	NA
1111294	3788.40	-4.35	1.26	0.001	0.044	Proteobacteria	Enterobacteriaceae	NA	NA
797229	31.92	-4.42	1.34	0.001	0.049	Proteobacteria	Enterobacteriaceae	NA	NA
New.ReferenceOTU968	20.73	-23.42	2.71	0.000	0.000	Spirochaetes	Spirochaetaceae	Treponema	NA
924224	85.85	-5.76	1.78	0.001	0.049	Spirochaetes	Spirochaetaceae	Treponema	NA
New.ReferenceOTU282	7.77	-4.35	1.29	0.001	0.049	Spirochaetes	Spirochaetaceae	Treponema	NA

Table S2. Differential abundance analysis of all measured microbial populations from caecal digesta of pigs fed either a control or laminarin supplemented diet

	OTU	Control	Lam	SEM	padj
**Phylum**					
Deferribacteres	4374042	0.21	0.08	0.09	0.620
Proteobacteria	113756	19.44	11.92	4.12	0.620
Bacteroidetes	568118	50.84	60.71	4.79	0.620
Firmicutes	New.CleanUp.ReferenceOTU122441	27.56	25.78	3.71	0.788
Actinobacteria	288683	0.07	0.10	0.02	0.788
Fibrobacteres	New.ReferenceOTU3654	0.04	0.06	0.03	0.788
Spirochaetes	68837	1.53	1.25	0.71	0.788
Fusobacteria	572889	0.20	0.00	0.09	0.873
Tenericutes	178759	0.10	0.10	0.05	0.873
**Family**					
Coriobacteriaceae	288683	0.07	0.11	0.02	0.692
RF16	New.ReferenceOTU3588	0.42	0.08	0.20	0.133
Prevotellaceae	568118	35.92	45.38	6.34	0.313
Paraprevotellaceae	20534	11.79	13.03	1.91	0.369
S24-7	577228	1.16	2.08	0.42	0.372
Odoribacteraceae	4307122	0.00	0.03	0.01	0.522
Porphyromonadaceae	921813	0.63	0.35	0.26	0.577
p-2534-18B5	16915	0.07	0.22	0.09	0.577
BS11	New.ReferenceOTU2170	0.04	0.01	0.02	0.801
Bacteroidaceae	New.ReferenceOTU2302	1.51	0.13	0.68	0.980
Deferribacteraceae	4374042	0.23	0.09	0.09	0.522
Fibrobacteraceae	New.ReferenceOTU3654	0.05	0.07	0.03	0.961
Streptococcaceae	349024	0.06	0.02	0.03	0.372
Peptococcaceae	New.ReferenceOTU2707	0.02	0.01	0.00	0.522
Clostridiaceae	215963	0.73	0.63	0.23	0.801
Ruminococcaceae	851865	7.27	10.37	1.87	0.801
Christensenellaceae	644244	0.02	0.05	0.02	0.961
Peptostreptococcaceae	712677	0.02	0.03	0.01	0.961
Lachnospiraceae	New.CleanUp.ReferenceOTU122441	12.76	10.09	2.27	0.980
Veillonellaceae	510572	2.20	1.25	0.53	0.980
[Mogibacteriaceae]	187517	0.09	0.12	0.04	0.980
Lactobacillaceae	302975	3.11	2.13	1.12	0.980
Turicibacteraceae	368490	0.19	0.05	0.09	0.980
Fusobacteriaceae	572889	0.24	0.00	0.11	0.980
Enterobacteriaceae	1111294	4.85	0.35	1.20	0.011
Alcaligenaceae	333380	0.15	0.06	0.03	0.243
Helicobacteraceae	548699	0.17	0.04	0.06	0.369
Desulfovibrionaceae	30569	0.10	0.26	0.07	0.372

Oxalobacteraceae	360508	0.00	0.01	0.00	0.719
Pasteurellaceae	359779	1.40	0.83	0.73	0.808
Campylobacteraceae	113756	11.39	9.88	3.44	0.961
Succinivibrionaceae	163857	1.59	0.82	0.79	0.980
Spirochaetaceae	68837	1.68	1.42	0.79	0.980
Anaeroplasmataceae	185593	0.11	0.01	0.03	0.980
**Genus**					
Collinsella	363794	0.03	0.04	0.01	0.791
Odoribacter	4307122	0.00	0.04	0.02	0.762
Prevotella	20534	11.99	13.22	2.14	0.791
YRC22	4435235	0.36	0.84	0.41	0.932
CF231	300853	1.88	1.60	0.53	0.932
Bacteroides	New.ReferenceOTU2302	1.95	0.17	0.89	0.932
Parabacteroides	921813	0.79	0.45	0.33	0.668
Paludibacter	New.ReferenceOTU1695	0.00	0.00	0.00	0.932
Prevotella	568118	42.75	51.58	6.66	0.091
Mucispirillum	4374042	0.27	0.10	0.11	0.791
Fibrobacter	New.ReferenceOTU3654	0.05	0.09	0.04	0.932
Anaerovorax	1112364	0.05	0.00	0.02	0.228
Clostridium	215963	0.41	0.30	0.14	0.932
Dorea	1076587	0.09	0.15	0.04	0.629
Blautia	532203	0.12	0.17	0.05	0.682
Roseburia	New.CleanUp.ReferenceOTU122441	5.23	3.32	1.65	0.932
Lachnospira	1029949	0.94	0.65	0.27	0.932
Coprococcus	344804	0.68	0.73	0.23	0.932
Butyrivibrio	4364564	0.08	0.01	0.03	0.932
Epulopiscium	New.ReferenceOTU2736	0.03	0.01	0.02	0.932
Ruminococcus	1111191	0.05	0.01	0.02	0.932
Anaerostipes	846386	0.01	0.02	0.01	0.932
Lactobacillus	302975	3.71	2.71	1.45	0.932
rc4-4	New.ReferenceOTU2707	0.02	0.01	0.01	0.629
Ruminococcus	369602	0.35	0.47	0.11	0.614
Faecalibacterium	851865	3.31	3.79	1.17	0.932
Oscillospira	310886	1.87	1.74	0.31	0.932
Streptococcus	349024	0.07	0.02	0.03	0.613
Turicibacter	368490	0.21	0.06	0.10	0.738
Dialister	264552	0.00	0.39	0.12	0.048
Acidaminococcus	25947	0.00	0.02	0.00	0.228
Anaerovibrio	510572	2.44	0.83	0.60	0.228
Megasphaera	266210	0.05	0.10	0.05	0.825
Phascolarctobacterium	916143	0.05	0.04	0.01	0.932
Mitsuokella	149335	0.08	0.08	0.04	0.932
Fusobacterium	572889	0.31	0.00	0.14	0.932
Sutterella	333380	0.18	0.08	0.03	0.435
Campylobacter	113756	14.02	12.05	4.34	0.932
Desulfovibrio	30569	0.08	0.32	0.08	0.613

Helicobacter	311173	0.06	0.05	0.02	0.932
Oxalobacter	360508	0.00	0.01	0.00	0.791
Aggregatibacter	9498	0.06	0.01	0.03	0.614
Actinobacillus	359779	1.51	0.98	0.80	0.932
Anaerobiospirillum	587570	0.03	0.06	0.03	0.791
Succinivibrio	163857	1.80	0.84	0.88	0.791
Treponema	68837	1.98	1.83	0.98	0.932
Anaeroplasma	New.ReferenceOTU3606	0.04	0.00	0.02	0.762
**Species**					
Lactobacillus mucosae	New.ReferenceOTU3048	0.05	0.07	0.05	0.780
Streptococcus luteciae	349024	0.13	0.05	0.07	0.780
Bacteroides plebeius	365496	0.18	0.03	0.09	0.780
Prevotella stercorea	591785	21.93	20.79	3.15	0.807
Prevotella copri	568118	64.56	61.44	5.64	0.893
Oxalobacter formigenes	360508	0.01	0.14	0.07	0.780
Ruminococcus gnavus	1111191	0.11	0.02	0.05	0.780
Coprococcus eutactus	362501	0.02	0.01	0.01	0.893
Dorea formicigenerans	1076587	0.09	0.28	0.11	0.807
Faecalibacterium prausnitzii	851865	11.55	15.65	4.81	0.780
Mitsuokella multacida	149335	0.17	0.13	0.07	0.893
Roseburia faecis	New.CleanUp.ReferenceOTU17239	0.07	0.03	0.02	0.807
Ruminococcus bromii	369602	0.16	0.71	0.27	0.301
Ruminococcus flavefaciens	New.ReferenceOTU3836	0.09	0.04	0.05	0.780
Collinsella aerofaciens	363794	0.07	0.13	0.03	0.780
Mucispirillum schaedleri	4374042	0.79	0.30	0.32	0.780
Fibrobacter succinogenes	638485	0.02	0.19	0.09	0.780

Table S3. Differential abundance analysis of the 3 significant OTUS from the colon pigs fed either a control or laminarin supplemented diet

OTU	baseMean	log2FoldChange	lfcSE	pvalue	padj	Kingdom	Phylum	Family	Genus	Species
New.ReferenceOTU968	85.12	-25.08	-9.13	6.8E-20	6.4E-17	Bacteria	Spirochaetales	Spirochaetaceae	Treponema	NA
879327	17.97	-23.40	-8.67	4.3E-18	2.0E-15	Bacteria	Clostridiales	NA	NA	NA
New.ReferenceOTU1384	201.24	6.30	4.05	5.1E-05	1.6E-02	Bacteria	Bacteroidales	Prevotellaceae	Prevotella	copri

Table S4. Differential abundance analysis of all measured microbial populations in the colonic digesta from pigs fed either a control or laminarin supplemented diet

	Control	Laminarin	SEM	padj
**Phylum**				
Actinobacteria	0.10	0.19	0.04	0.655
Spirochaetes	2.79	2.47	0.80	0.655
Deferribacteres	0.69	0.31	0.26	0.655
Proteobacteria	12.79	7.56	3.10	0.655
Bacteroidetes	52.09	50.24	4.06	0.655
Fibrobacteres	0.10	0.12	0.04	0.826
Fusobacteria	0.35	0.00	0.17	0.826
Firmicutes	30.88	38.82	4.63	0.826
Tenericutes	0.20	0.29	0.08	0.931
**Family**				
Coriobacteriaceae	0.11	0.22	0.04	0.672
p-2534-18B5	1.15	0.10	0.33	0.033
Porphyromonadaceae	1.21	0.64	0.30	0.357
BS11	0.21	0.02	0.06	0.359
S24-7	2.73	4.44	1.03	0.603
Prevotellaceae	30.35	33.43	5.03	0.708
[Odoribacteraceae]	0.00	0.03	0.01	0.741
[Paraprevotellaceae]	13.77	11.38	1.46	0.765
RF16	0.88	0.18	0.37	0.971
Bacteroidaceae	2.53	0.30	1.12	0.981
Deferribacteraceae	0.83	0.34	0.31	0.825
Fibrobacteraceae	0.12	0.13	0.05	0.971
Christensenellaceae	1.72	0.82	0.77	0.197
Lachnospiraceae	12.99	13.45	1.89	0.603
Ruminococcaceae	11.49	18.45	3.01	0.603
Veillonellaceae	1.12	1.10	0.30	0.741
Mogibacteriaceae	0.22	0.19	0.07	0.848
Clostridiaceae	1.02	1.22	0.30	0.858
Peptostreptococcaceae	0.02	0.03	0.02	0.955
Peptococcaceae	0.04	0.04	0.01	0.971
Lactobacillaceae	1.79	2.77	0.55	0.708
Streptococcaceae	0.03	0.02	0.02	0.808

Turicibacteraceae	0.05	0.04	0.03	0.765
Fusobacteriaceae	0.41	0.00	0.19	0.848
Succinivibrionaceae	0.59	0.49	0.33	0.765
Alcaligenaceae	0.16	0.24	0.05	0.708
Oxalobacteraceae	0.02	0.02	0.01	0.825
Helicobacteraceae	1.23	0.28	0.55	0.305
Campylobacteraceae	5.61	4.72	1.99	0.708
Desulfovibrionaceae	0.35	0.37	0.11	0.971
Enterobacteriaceae	3.73	0.38	0.95	0.305
Pasteurellaceae	0.06	1.16	0.50	0.858
Spirochaetaceae	3.28	2.84	0.93	0.848
Anaeroplasmataceae	0.17	0.17	0.08	0.963
**Genus**				
Anaerovorax	0.05	0.14	0.05	0.975
Acidaminococcus	0.00	0.03	0.02	0.975
YRC22	0.55	1.42	0.69	0.357
Prevotella	15.06	10.43	1.72	0.357
Parabacteroides	3.62	3.53	1.00	0.975
Epulopiscium	3.48	0.43	1.53	0.975
Campylobacter	1.62	0.89	0.42	0.360
Lachnospira	0.09	0.01	0.03	0.975
Dorea	40.97	43.03	5.18	0.975
Butyrivibrio	1.09	0.43	0.40	0.975
Collinsella	0.17	0.18	0.07	0.981
Treponema	0.12	0.00	0.05	0.000
Anaerobiospirillum	0.65	0.50	0.31	0.975
Succinivibrio	0.02	0.00	0.01	0.425
Aggregatibacter	0.68	0.53	0.17	0.742
Actinobacillus	0.12	0.30	0.06	0.742
Sutterella	0.02	0.01	0.01	0.975
Oxalobacter	0.01	0.03	0.01	0.975
Mucispirillum	1.05	1.13	0.22	0.975
Bacteroides	0.02	0.03	0.01	0.975
Prevotella	3.78	4.96	1.34	0.979
CF231	0.32	0.40	0.11	0.979
Odoribacter	2.57	3.94	0.82	0.975
Paludibacter	0.06	0.05	0.02	0.975
Fusobacterium	3.11	7.39	2.11	0.975

Anaeroplasma	1.42	1.99	0.63	0.975
Turicibacter	2.85	2.84	0.49	0.975
Streptococcus	0.04	0.03	0.02	0.975
Lactobacillus	0.09	0.05	0.05	0.975
Phascolarctobacterium	0.00	0.01	0.00	0.114
Mitsuokella	0.05	0.06	0.01	0.975
Anaerovibrio	0.04	0.09	0.05	0.975
Dialister	1.37	1.06	0.34	0.975
Clostridium	0.00	0.13	0.06	0.975
rc4-4	0.02	0.03	0.01	0.986
Faecalibacterium	0.57	0.00	0.27	0.975
Ruminococcus	0.22	0.31	0.06	0.975
Oscillospira	7.44	6.37	2.63	0.510
[Ruminococcus]	0.41	0.46	0.15	0.979
Coprococcus	0.28	0.38	0.09	0.981
Anaerostipes	0.02	0.03	0.01	0.975
Roseburia	0.00	0.06	0.03	0.975
Blautia	0.09	1.49	0.64	0.975
Desulfovibrio	0.05	0.09	0.05	0.975
Fibrobacter	0.78	0.53	0.43	0.975
Helicobacter	4.88	3.99	1.38	0.975
Megasphaera	0.11	0.20	0.11	0.975
**Species**				
Bacteroides plebius	0.01	0.19	0.08	0.310
Coprococcus eutactus	0.02	0.01	0.01	0.701
Dorea formicigenerans	0.09	0.26	0.10	0.701
Collinsella aerofaciens	0.12	0.08	0.03	0.701
Fibrobacter succinogenes	0.03	0.16	0.08	0.701
Oxalobacter formigenes	0.01	0.13	0.06	0.701
Prevotella stercorea	21.85	20.98	3.12	0.701
Prevotella copri	65.36	61.08	5.70	0.701
Streptococcus luteciae	0.14	0.05	0.08	0.701
Lactobacillus mucosae	0.06	0.06	0.05	0.701
Faecalibacterium prausnitzii	10.55	16.08	4.53	0.701
Ruminococcus gnavus	0.11	0.03	0.05	0.701
Ruminococcus flavefaciens	0.11	0.03	0.05	0.731

Mucispirillum schaedleri	1.02	0.14	0.28	0.830
Mitsuokella multacida	0.16	0.14	0.07	0.830
Ruminococcus bromii	0.29	0.53	0.27	0.830
Roseburia faecis	0.07	0.04	0.02	0.847

Table S5. Differential analysis of bacterial functionality at levels 1-3 and KO terms based on predicted functionality using PICRUSt

Function	baseMean	log2Fold Change	lfcSE	p
Metabolism; Other	28745430.69	0.03	0.01	
Genetic Information Processing; Other	13131827.07	0.04	0.02	
Organismal Systems; Other	488655.04	0.10	0.06	
Environmental Information Processing; Other	6681649.74	-0.10	0.10	
Human Diseases; Other	532802.23	-0.07	0.07	
None; Other	115763.06	0.05	0.05	
Cellular Processes; Other	1947519.41	-0.08	0.13	
Unclassified; Other	8303698.82	-0.01	0.01	
L2				
Cellular Processes;Cell Growth and Death	356127.74	0.05	0.04	
Cellular Processes;Transport and Catabolism	187020.82	0.09	0.07	
Environmental Information Processing;Signal Transduction	829923.90	-0.17	0.14	
Environmental Information Processing;Signaling Molecules and Interaction	111185.82	0.07	0.06	
Genetic Information Processing;Replication and Repair	6045000.30	0.04	0.03	
Genetic Information Processing;Translation	3901920.81	0.04	0.02	
Human Diseases;Infectious Diseases	254850.52	-0.06	0.04	
Metabolism;Biosynthesis of Other Secondary Metabolites	558988.33	0.08	0.06	
Metabolism;Enzyme Families	1374962.67	0.04	0.03	
Metabolism;Nucleotide Metabolism	2796574.19	0.04	0.03	
Organismal Systems;Endocrine System	190408.74	0.12	0.08	
Organismal Systems;Immune System	64313.27	0.08	0.06	
Organismal Systems;Nervous System	56149.65	0.10	0.08	
Unclassified;Cellular Processes and Signaling	2409232.51	-0.04	0.03	
Environmental Information Processing;Membrane Transport	5755765.15	-0.11	0.11	
Human Diseases;Metabolic Diseases	68084.49	0.03	0.03	
Organismal Systems;Digestive System	61839.93	0.22	0.23	
Unclassified;Poorly Characterized	2891044.99	-0.02	0.02	
Cellular Processes;Cell Motility	1409065.42	-0.15	0.19	
Human Diseases;Cancers	76858.56	-0.05	0.07	
Human Diseases;Cardiovascular Diseases	10.59	1.34	2.02	
Human Diseases;Neurodegenerative Diseases	101793.38	-0.28	0.35	
Metabolism;Metabolism of Terpenoids and Polyketides	1073657.00	0.03	0.03	
Metabolism;Xenobiotics Biodegradation and Metabolism	808595.11	-0.04	0.06	
Organismal Systems;Circulatory System	11547.01	-0.57	0.77	
Unclassified;Genetic Information Processing	1634557.09	-0.02	0.03	
Genetic Information Processing;Folding, Sorting and Degradation	1695035.37	0.00	0.03	

Genetic Information Processing;Transcription	1485966.72	0.01	0.05	0
Human Diseases;Immune System Diseases	31721.42	-0.02	0.07	0
Metabolism;Amino Acid Metabolism	5573293.90	0.01	0.03	0
Metabolism;Carbohydrate Metabolism	5686094.15	0.02	0.04	0
Metabolism;Energy Metabolism	3749124.62	0.01	0.02	0
Metabolism;Glycan Biosynthesis and Metabolism	1867964.13	0.02	0.07	0
Metabolism;Lipid Metabolism	1537993.45	-0.01	0.04	0
Metabolism;Metabolism of Cofactors and Vitamins	2782637.48	0.01	0.03	0
Metabolism;Metabolism of Other Amino Acids	933880.95	0.00	0.02	0
Organismal Systems;Environmental Adaptation	98663.94	0.01	0.07	0
Organismal Systems;Excretory System	5273.48	0.08	0.41	0
Unclassified;Metabolism	1371127.06	-0.01	0.03	0
L3				
Cellular Processes;Cell Growth and Death	356104.30	0.05	0.04	0
Cellular Processes;Transport and Catabolism	187009.94	0.09	0.07	0
Environmental Information Processing;Signal Transduction	829861.09	-0.17	0.14	0
Environmental Information Processing;Signaling Molecules and Interaction	111179.12	0.07	0.06	0
Genetic Information Processing;Replication and Repair	6044610.97	0.04	0.03	0
Genetic Information Processing;Translation	3901667.46	0.04	0.02	0
Human Diseases;Infectious Diseases	254834.82	-0.06	0.04	0
Metabolism;Biosynthesis of Other Secondary Metabolites	558953.02	0.08	0.06	0
Metabolism;Enzyme Families	1374875.45	0.04	0.03	0
Metabolism;Nucleotide Metabolism	2796395.62	0.04	0.03	0
Organismal Systems;Endocrine System	190397.30	0.12	0.08	0
Organismal Systems;Immune System	64308.94	0.08	0.06	0
Organismal Systems;Nervous System	56146.36	0.10	0.08	0
Unclassified;Cellular Processes and Signaling	2409075.05	-0.04	0.03	0
Human Diseases;Metabolic Diseases	68080.23	0.03	0.03	0
Environmental Information Processing;Membrane Transport	5755336.53	-0.11	0.11	0
Organismal Systems;Digestive System	61837.00	0.22	0.23	0
Unclassified;Poorly Characterized	2890857.74	-0.02	0.02	0
Human Diseases;Neurodegenerative Diseases	101784.28	-0.28	0.31	0
Metabolism;Metabolism of Terpenoids and Polyketides	1073588.02	0.03	0.03	0
Cellular Processes;Cell Motility	1408947.88	-0.15	0.19	0
Human Diseases;Cardiovascular Diseases	10.59	1.34	1.87	0
Metabolism;Xenobiotics Biodegradation and Metabolism	808539.10	-0.04	0.06	0
Organismal Systems;Circulatory System	11545.64	-0.57	0.78	0
Human Diseases;Cancers	76853.63	-0.05	0.07	0
Unclassified;Genetic Information Processing	1584636.87	-0.02	0.03	0
Metabolism;Metabolism of Cofactors and Vitamins	2782460.03	0.01	0.03	0
Genetic Information Processing;Transcription	1485867.12	0.01	0.05	0
Human Diseases;Immune System Diseases	31719.29	-0.02	0.07	0
Metabolism;Amino Acid Metabolism	5572916.87	0.01	0.03	0

Metabolism;Carbohydrate Metabolism	5685716.86	0.02	0.04	0
Metabolism;Energy Metabolism	3748876.01	0.01	0.02	0
Metabolism;Glycan Biosynthesis and Metabolism	1867853.27	0.02	0.07	0
Metabolism;Lipid Metabolism	1537891.11	-0.01	0.04	0
Metabolism;Metabolism of Other Amino Acids	933820.75	0.00	0.02	0
Organismal Systems;Environmental Adaptation	98656.88	0.01	0.07	0
Organismal Systems;Excretory System	5273.17	0.08	0.41	0
Unclassified;Metabolism	1420851.41	-0.01	0.04	0
Genetic Information Processing;Folding, Sorting and Degradation	1694926.07	0.00	0.03	0
KO				
K07445	55.42	23.95	2.96	0
K01041	242.62	-3.52	0.73	0
K02532	645.69	-2.68	0.57	0
K01085	631.06	-2.68	0.60	0
K05591	426.73	-3.94	1.24	0
K05596	432.56	-3.86	1.08	0
K02457	444.17	-3.46	1.00	0
K02452	528.06	-3.00	0.95	0
K02458	444.17	-3.46	1.00	0
K02459	444.17	-3.46	1.00	0
K08137	426.73	-3.94	1.24	0
K13014	426.73	-3.94	1.24	0
K08567	722.41	-2.43	0.62	0
K03275	426.73	-3.94	1.24	0
K09904	476.25	-3.12	1.00	0
K01826	422.10	-3.89	1.24	0
K01825	1939.61	-3.28	0.98	0
K02849	525.59	-3.22	0.83	0
K14061	1512.54	-3.13	0.97	0
K14062	426.73	-3.94	1.24	0
K02844	522.82	-3.16	1.00	0
K02847	426.73	-3.94	1.24	0
K07349	853.91	-3.93	1.15	0
K07348	426.73	-3.94	1.24	0
K07345	2788.60	-3.45	0.99	0
K07347	3027.62	-3.12	0.96	0
K07346	1513.79	-3.12	0.97	0
K07340	476.25	-3.12	1.00	0
K08993	494.39	-3.52	0.95	0
K08344	420.80	-3.95	1.24	0
K05921	1118.46	-2.82	0.89	0
K05820	476.25	-3.12	1.00	0
K02478	458.03	-3.47	0.94	0
K11748	440.26	-3.56	1.04	0
K11742	1143.53	-2.11	0.67	0

K09923	476.25	-3.12	1.00	0
K01669	582.24	-2.74	0.82	0
K12152	446.40	-3.56	1.04	0
K00322	426.73	-3.94	1.24	0
K10804	857.23	-3.76	1.12	0
K10805	446.40	-3.56	1.04	0
K05804	1645.06	-2.54	0.79	0
K05805	452.97	-3.56	0.96	0
K05800	430.82	-3.88	1.19	0
K00121	1092.89	-2.54	0.67	0
K13051	433.81	-3.82	1.08	0
K12058	444.79	-3.46	0.99	0
K06140	426.73	-3.94	1.24	0
K02082	524.05	-2.62	0.72	0
K03933	426.73	-3.94	1.24	0
K02336	428.09	-3.89	1.24	0
K07306	1165.64	-2.46	0.78	0
K02610	420.80	-3.95	1.24	0
K02612	420.80	-3.95	1.24	0
K09476	420.80	-3.95	1.24	0
K09471	476.25	-3.12	1.00	0
K06219	426.73	-3.94	1.24	0
K08485	426.73	-3.94	1.24	0
K08484	450.30	-3.46	1.00	0
K03919	422.10	-3.89	1.24	0
K08163	420.80	-3.95	1.24	0
K08359	433.04	-3.58	1.06	0
K08354	420.80	-3.95	1.24	0
K08350	469.92	-3.13	0.95	0
K00855	426.73	-3.94	1.24	0
K03767	585.72	-2.60	0.73	0
K03762	480.37	-2.88	0.86	0
K12582	456.80	-3.36	1.00	0
K03970	432.56	-3.86	1.08	0
K03974	432.56	-3.86	1.08	0
K05517	431.15	-3.97	1.23	0
K07700	426.73	-3.94	1.24	0
K07702	426.73	-3.94	1.24	0
K07708	452.97	-3.56	0.96	0
K06212	530.47	-2.42	0.74	0
K02618	420.80	-3.95	1.24	0
K02616	420.80	-3.95	1.24	0
K02611	420.80	-3.95	1.24	0
K02613	420.80	-3.95	1.24	0
K06866	476.25	-3.12	1.00	0
K14089	351.07	1.94	0.52	0

K01554	420.80	-3.95	1.24	0
K02399	428.09	-3.89	1.24	0
K02391	428.09	-3.89	1.24	0
K12339	452.97	-3.56	0.96	0
K14060	420.80	-3.95	1.24	0
K00632	1554.58	-1.97	0.57	0
K07810	445.52	-3.60	0.96	0
K00892	426.73	-3.94	1.24	0
K08151	465.66	-3.23	0.81	0
K08154	426.73	-3.94	1.24	0
K01761	562.10	-2.69	0.76	0
K01766	476.25	-3.12	1.00	0
K08167	426.73	-3.94	1.24	0
K14414	420.80	-3.95	1.24	0
K03721	486.09	-3.03	0.91	0
K00151	420.80	-3.95	1.24	0
K04091	423.00	-3.90	1.15	0
K02001	625.94	-2.64	0.71	0
K02000	688.62	-2.59	0.79	0
K02002	707.77	-2.49	0.73	0
K07746	433.04	-3.58	1.06	0
K10778	434.75	-3.74	1.08	0
K01906	66.52	4.46	1.31	0
K09921	447.70	-3.53	1.04	0
K10906	420.80	-3.95	1.24	0
K04058	420.80	-3.95	1.24	0
K01460	446.40	-3.56	1.04	0
K11192	426.73	-3.94	1.24	0
K10716	426.73	-3.94	1.24	0
K07783	677.06	-2.44	0.75	0
K01252	420.80	-3.95	1.24	0
K01070	435.41	-3.87	1.16	0
K01782	1844.57	-2.48	0.71	0
K03782	477.50	-2.95	0.88	0
K03788	456.80	-3.36	1.00	0
K09788	655.11	-2.22	0.66	0
K06144	426.73	-3.94	1.24	0
K02565	438.53	-3.98	1.24	0
K02508	420.80	-3.95	1.24	0
K06080	426.73	-3.94	1.24	0
K06887	1512.54	-3.13	0.97	0
K00483	583.08	-2.29	0.69	0
K00484	440.38	-3.54	1.06	0
K09901	476.25	-3.12	1.00	0
K07684	476.33	-3.35	0.89	0
K07686	652.09	-2.48	0.78	0

K07689	426.73	-3.94	1.24	0
K04016	476.25	-3.12	1.00	0
K12972	855.71	-3.84	1.13	0
K12973	428.09	-3.89	1.24	0
K12974	426.73	-3.94	1.24	0
K12288	426.73	-3.94	1.24	0
K12289	426.73	-3.94	1.24	0
K00073	460.59	-3.19	0.96	0
K08276	433.44	-3.78	1.08	0
K02746	683.99	-2.08	0.58	0
K02747	498.44	-3.10	0.78	0
K02745	527.95	-3.00	0.73	0
K07666	476.25	-3.12	1.00	0
K07664	426.73	-3.94	1.24	0
K07660	426.73	-3.94	1.24	0
K07002	1444.82	-1.76	0.55	0
K09181	706.22	-2.62	0.70	0
K01150	458.41	-3.41	0.95	0
K05522	420.80	-3.95	1.24	0
K11933	434.11	-3.92	1.16	0
K11932	426.73	-3.94	1.24	0
K11939	426.73	-3.94	1.24	0
K07274	460.36	-4.00	1.23	0
K02485	450.30	-3.46	1.00	0
K02487	420.80	-3.95	1.24	0
K09954	426.73	-3.94	1.24	0
K07490	426.73	-3.94	1.24	0
K11203	637.56	-2.11	0.59	0
K11201	420.80	-3.95	1.24	0
K14187	1244.95	-2.39	0.74	0
K02769	909.08	-1.48	0.45	0
K09895	476.25	-3.12	1.00	0
K09894	476.25	-3.12	1.00	0
K03828	443.67	-3.69	1.05	0
K03824	432.56	-3.86	1.08	0
K11911	426.73	-3.94	1.24	0
K12062	420.80	-3.95	1.24	0
K12066	420.80	-3.95	1.24	0
K07251	456.80	-3.36	1.00	0
K09975	426.73	-3.94	1.24	0
K09978	426.73	-3.94	1.24	0
K11263	420.80	-3.95	1.24	0
K13053	426.73	-3.94	1.24	0
K08369	711.23	-1.93	0.56	0
K06937	183.92	1.45	0.44	0
K05916	451.77	-3.60	0.96	0

K05875	426.73	-3.94	1.24	0
K05874	428.09	-3.89	1.24	0
K05877	426.73	-3.94	1.24	0
K03840	426.73	-3.94	1.24	0
K09910	476.25	-3.12	1.00	0
K09916	426.73	-3.94	1.24	0
K09917	476.25	-3.12	1.00	0
K11535	426.73	-3.94	1.24	0
K11537	523.45	-3.87	0.98	0
K01584	428.09	-3.89	1.24	0
K08348	452.45	-3.33	0.99	0
K01638	436.54	-3.80	1.07	0
K01637	428.09	-3.89	1.24	0
K02615	420.80	-3.95	1.24	0
K02852	538.69	-2.72	0.85	0
K02853	456.80	-3.36	1.00	0
K03675	440.31	-3.57	1.04	0
K06006	426.73	-3.94	1.24	0
K07357	847.87	-3.94	1.15	0
K07350	426.73	-3.94	1.24	0
K07351	426.73	-3.94	1.24	0
K06918	476.25	-3.12	1.00	0
K06916	536.14	-2.99	0.93	0
K09161	446.40	-3.56	1.04	0
K09160	476.25	-3.12	1.00	0
K01354	426.73	-3.94	1.24	0
K01355	420.80	-3.95	1.24	0
K03623	455.27	-3.41	0.91	0
K03212	476.25	-3.12	1.00	0
K07136	426.73	-3.94	1.24	0
K13301	426.73	-3.94	1.24	0
K07165	422.10	-3.89	1.24	0
K07160	1724.78	-1.12	0.35	0
K03472	426.73	-3.94	1.24	0
K06970	431.15	-3.97	1.23	0
K05836	426.73	-3.94	1.24	0
K05839	426.73	-3.94	1.24	0
K07677	426.73	-3.94	1.24	0
K02464	426.08	-3.87	1.13	0
K02462	420.80	-3.95	1.24	0
K02461	420.80	-3.95	1.24	0
K02460	528.06	-3.00	0.95	0
K03225	433.81	-3.82	1.08	0
K08682	426.73	-3.94	1.24	0
K11750	426.73	-3.94	1.24	0
K13938	420.80	-3.95	1.24	0

K07146	498.05	-3.30	0.81	0
K07140	458.15	-3.33	1.00	0
K08308	426.73	-3.94	1.24	0
K12149	456.80	-3.36	1.00	0
K12148	426.73	-3.94	1.24	0
K07312	426.73	-3.94	1.24	0
K06956	1513.35	-1.77	0.50	0
K05812	426.73	-3.94	1.24	0
K05811	426.73	-3.94	1.24	0
K05816	438.80	-3.49	0.97	0
K01146	426.73	-3.94	1.24	0
K02403	428.09	-3.89	1.24	0
K02402	428.09	-3.89	1.24	0
K13041	457.86	-3.31	0.90	0
K13040	433.04	-3.58	1.06	0
K07120	474.23	-3.57	0.94	0
K01690	428.09	-3.89	1.24	0
K03435	446.40	-3.56	1.04	0
K02345	426.73	-3.94	1.24	0
K05997	426.73	-3.94	1.24	0
K03812	426.73	-3.94	1.24	0
K01194	420.80	-3.95	1.24	0
K05343	1113.20	1.85	0.58	0
K02425	426.73	-3.94	1.24	0
K03776	428.09	-3.89	1.24	0
K03777	456.80	-3.36	1.00	0
K08478	420.80	-3.95	1.24	0
K08476	420.80	-3.95	1.24	0
K08475	420.80	-3.95	1.24	0
K00455	420.80	-3.95	1.24	0
K04752	430.82	-3.88	1.19	0
K02363	426.73	-3.94	1.24	0
K11103	463.43	-3.39	0.91	0
K06445	428.09	-3.89	1.24	0
K07642	462.56	-3.01	0.92	0
K07394	426.73	-3.94	1.24	0
K04103	432.56	-3.86	1.08	0
K07715	539.55	-2.98	0.93	0
K07711	426.73	-3.94	1.24	0
K13771	565.40	-2.81	0.87	0
K02198	922.64	-1.71	0.50	0
K02193	782.38	-1.89	0.52	0
K02197	896.95	-1.73	0.51	0
K02194	896.95	-1.73	0.51	0
K03717	451.60	-3.43	1.00	0
K11733	426.73	-3.94	1.24	0

K07806	426.73	-3.94	1.24	0
K02609	420.80	-3.95	1.24	0
K00146	420.80	-3.95	1.24	0
K14090	397.67	1.82	0.46	0
K02848	426.73	-3.94	1.24	0
K06222	420.80	-3.95	1.24	0
K07285	469.71	-3.20	0.89	0
K00883	497.49	-2.80	0.81	0
K08172	420.80	-3.95	1.24	0
K12684	452.45	-3.33	0.99	0
K12685	476.33	-3.35	0.89	0
K04080	426.73	-3.94	1.24	0
K10748	426.73	-3.94	1.24	0
K07771	426.73	-3.94	1.24	0
K12518	847.87	-3.94	1.15	0
K12519	847.87	-3.94	1.15	0
K12517	847.87	-3.94	1.15	0
K07186	476.25	-3.12	1.00	0
K04062	441.61	-3.53	1.04	0
K10004	452.97	-3.56	0.96	0
K10002	452.97	-3.56	0.96	0
K10003	452.97	-3.56	0.96	0
K10001	455.62	-3.49	0.96	0
K14588	426.73	-3.94	1.24	0
K02423	426.73	-3.94	1.24	0
K11183	445.97	-3.51	0.97	0
K07796	436.87	-3.90	1.08	0
K07795	1186.43	-2.05	0.60	0
K00228	452.11	-3.45	1.00	0
K01061	453.53	-3.22	1.00	0
K12530	426.73	-3.94	1.24	0
K12531	420.80	-3.95	1.24	0
K12532	420.80	-3.95	1.24	0
K04046	428.09	-3.89	1.24	0
K05790	446.40	-3.56	1.04	0
K05791	426.73	-3.94	1.24	0
K05792	461.62	-3.19	0.86	0
K05793	426.73	-3.94	1.24	0
K05798	426.73	-3.94	1.24	0
K01479	424.96	-3.82	1.13	0
K02079	420.80	-3.95	1.24	0
K06175	499.47	-2.87	0.87	0
K06858	426.73	-3.94	1.24	0
K07229	426.73	-3.94	1.24	0
K07225	426.73	-3.94	1.24	0
K07454	420.80	-3.95	1.24	0

K08227	426.73	-3.94	1.24	0
K02790	420.80	-3.95	1.24	0
K03112	426.73	-3.94	1.24	0
K07054	674.67	-2.36	0.73	0
K13638	426.73	-3.94	1.24	0
K13639	426.73	-3.94	1.24	0
K00108	434.85	-3.82	1.18	0
K06156	426.73	-3.94	1.24	0
K11940	426.73	-3.94	1.24	0
K02511	420.80	-3.95	1.24	0
K07070	426.73	-3.94	1.24	0
K12963	426.73	-3.94	1.24	0
K12961	426.73	-3.94	1.24	0
K14744	1512.54	-3.13	0.97	0
K02167	426.73	-3.94	1.24	0
K11923	434.11	-3.92	1.16	0
K11926	426.73	-3.94	1.24	0
K11925	426.73	-3.94	1.24	0
K07269	426.73	-3.94	1.24	0
K07480	2787.25	-3.45	0.99	0
K07489	847.87	-3.94	1.15	0
K00932	420.80	-3.95	1.24	0
K03485	428.12	-3.92	1.16	0
K07675	894.03	-2.03	0.56	0
K07676	426.73	-3.94	1.24	0
K07673	451.77	-3.60	0.96	0
K05880	420.80	-3.95	1.24	0
K05881	440.31	-3.57	1.04	0
K06195	428.09	-3.89	1.24	0
K11909	426.73	-3.94	1.24	0
K11906	428.99	-3.90	1.15	0
K11903	966.17	-3.04	0.93	0
K07246	1693.31	-1.05	0.33	0
K01601	58.52	6.94	1.93	0
K03603	476.25	-3.12	1.00	0
K06078	426.73	-3.94	1.24	0
K06079	480.17	-3.24	0.93	0
K06073	426.73	-3.94	1.24	0
K06074	426.73	-3.94	1.24	0
K07659	560.95	-2.85	0.83	0
K09912	420.80	-3.95	1.24	0
K09914	432.56	-3.86	1.08	0
K11250	451.77	-3.60	0.96	0
K11258	476.25	-3.12	1.00	0
K10124	420.80	-3.95	1.24	0
K03668	451.77	-3.60	0.96	0

K09771	448.11	-3.43	0.92	0
K07637	426.73	-3.94	1.24	0
K07638	476.98	-3.22	0.88	0
K05903	275.29	2.81	0.72	0
K01500	470.52	1.41	0.45	0
K11904	1583.27	-2.76	0.88	0
K03774	579.84	-2.43	0.78	0
K08319	470.09	-3.12	1.00	0
K09890	470.09	-3.12	1.00	0
K09900	469.08	-3.12	1.00	0
K00884	477.60	-3.10	0.99	0
K01468	823.61	-1.67	0.54	0
K05501	721.30	-2.22	0.71	0
K04097	423.39	-3.85	1.24	0
K07774	423.39	-3.85	1.24	0
K06039	660.12	-2.44	0.79	0
K00219	643.29	-1.98	0.64	0
K07685	660.12	-2.44	0.79	0
K07644	429.38	-3.85	1.24	0
K10680	429.38	-3.85	1.24	0
K04067	660.12	-2.44	0.79	0
K00371	759.62	-1.69	0.55	0
K00370	759.62	-1.69	0.55	0
K00373	759.62	-1.69	0.55	0
K02552	715.98	-2.13	0.69	0
K04014	617.45	-2.26	0.73	0
K12940	605.07	-2.09	0.68	0
K13695	1284.67	-2.03	0.66	0
K12369	720.66	-2.09	0.68	0
K08680	725.60	-2.29	0.75	0
K07733	1127.60	-2.69	0.88	0
K01066	709.40	-2.00	0.65	0
K08994	427.53	-3.70	1.20	0
K07156	433.50	-3.69	1.20	0
K06946	433.50	-3.69	1.20	0
K07245	433.50	-3.69	1.20	0
K02572	763.68	-2.24	0.73	0
K03566	482.69	-3.00	0.98	0
K09795	1024.73	-2.40	0.79	0
K01664	1419.55	-1.79	0.59	0
K01720	1020.28	-2.41	0.79	0
K07649	425.94	-3.76	1.24	0
K03417	1021.47	-2.40	0.79	0
K12056	1015.65	-2.40	0.79	0
K01011	2893.21	-1.45	0.48	0
K10011	1091.64	-2.90	0.96	0

K11996	669.38	-2.17	0.72	0
K07000	778.55	-2.26	0.75	0
K12068	1013.09	-2.40	0.79	0
K12063	1013.09	-2.40	0.79	0
K12060	1013.09	-2.40	0.79	0
K12061	1013.09	-2.40	0.79	0
K12065	1013.09	-2.40	0.79	0
K05368	1091.64	-2.90	0.96	0
K12525	1085.47	-2.89	0.96	0
K01160	1085.47	-2.89	0.96	0
K07264	1169.43	-2.76	0.91	0
K03837	1581.44	-1.62	0.53	0
K07275	667.34	-2.35	0.78	0
K07334	1379.04	-1.34	0.45	0
K01407	670.28	-2.37	0.79	0
K07712	672.80	-2.60	0.87	0
K14260	3829.70	-0.89	0.30	0
K02430	664.27	-2.37	0.79	0
K00313	560.19	-2.15	0.72	0
K04013	590.99	-2.46	0.83	0
K12057	1037.10	-2.33	0.78	0
K12067	1037.10	-2.33	0.78	0
K08996	546.42	-2.14	0.72	0
K00758	450.40	-3.20	1.08	0
K02445	1461.50	-1.00	0.34	0
K08325	521.90	-2.96	1.00	0
K07482	953.28	1.95	0.66	0
K10679	745.71	-2.24	0.76	0
K06211	663.50	-1.79	0.61	0
K07286	696.38	-2.26	0.77	0
K13926	745.30	-2.30	0.78	0
K14392	1351.40	-1.35	0.46	0
K05982	510.73	-3.27	1.12	0
K01792	489.89	-2.90	0.99	0
K03297	1723.44	-1.56	0.53	0
K11896	1141.35	-2.53	0.87	0
K03746	765.02	-2.11	0.73	0
K13256	609.34	-1.92	0.66	0
K05785	592.62	-2.71	0.94	0
K02688	651.49	-1.84	0.64	0
K07308	533.29	-2.25	0.78	0
K02553	701.44	-2.31	0.80	0
K07034	798.21	-2.21	0.77	0
K09907	689.79	-2.29	0.80	0
K08990	689.79	-2.29	0.80	0
K12372	689.79	-2.29	0.80	0

K12370	689.79	-2.29	0.80	0
K12371	689.79	-2.29	0.80	0
K09896	689.79	-2.29	0.80	0
K09898	689.79	-2.29	0.80	0
K07640	689.79	-2.29	0.80	0
K05952	689.79	-2.29	0.80	0
K03214	689.79	-2.29	0.80	0
K11900	544.17	-2.57	0.90	0
K11901	544.17	-2.57	0.90	0
K06906	544.10	-2.56	0.89	0
K02472	678.95	-2.28	0.80	0
K02569	774.26	-1.85	0.65	0
K07784	683.74	-2.28	0.80	0
K08159	830.38	-1.98	0.69	0
K08138	237.82	-2.24	0.79	0
K07109	685.70	-2.25	0.79	0
K06282	991.30	-1.59	0.56	0
K06281	991.30	-1.59	0.56	0
K01141	750.35	-2.19	0.77	0
K02339	742.78	-2.16	0.77	0
K07694	375.48	1.34	0.48	0
K03926	579.31	-2.36	0.84	0
K11907	543.27	-2.57	0.91	0
K00344	725.93	-2.14	0.76	0
K03560	740.04	-2.16	0.77	0
K04772	740.04	-2.16	0.77	0
K10914	1371.57	-1.39	0.50	0
K09862	740.04	-2.16	0.77	0
K01247	728.44	-1.61	0.58	0
K07014	741.83	-2.14	0.77	0
K06203	768.93	-2.10	0.76	0
K13889	665.70	-1.72	0.62	0
K02200	861.59	-1.88	0.67	0
K03567	588.60	-2.37	0.86	0
K03684	1050.42	-1.58	0.57	0
K02742	773.78	-2.19	0.80	0
K02052	2176.93	-1.08	0.39	0
K11751	1614.50	-2.05	0.74	0
K06904	963.39	-1.33	0.48	0
K09858	1005.71	-1.73	0.63	0
K02301	695.54	-2.31	0.85	0
K07019	541.08	-2.40	0.88	0
K02784	962.36	-1.92	0.70	0
K03756	238.65	2.23	0.82	0
K03583	1066.45	-1.78	0.66	0
K14393	1688.44	-1.80	0.67	0

K06077	904.64	-1.99	0.74	0
K06879	956.77	-1.86	0.69	0
K06968	696.03	-2.28	0.85	0
K01139	2169.13	-1.22	0.45	0
K00060	755.94	-1.54	0.57	0
K00568	1721.10	-1.90	0.71	0
K02195	693.76	-1.87	0.70	0
K09925	215.76	-1.41	0.53	0
K03808	1924.54	-1.88	0.71	0
K07396	499.50	-2.42	0.91	0
K07250	1508.46	-1.04	0.39	0
K14055	684.30	-2.36	0.89	0
K03314	684.30	-2.36	0.89	0
K11107	684.30	-2.36	0.89	0
K02439	1002.96	-1.33	0.50	0
K03185	732.01	-2.12	0.80	0
K04085	1006.80	-1.77	0.67	0
K02116	1723.60	-1.04	0.39	0
K02168	1640.75	-2.10	0.80	0
K06886	714.05	-1.67	0.63	0
K03222	671.52	-2.42	0.92	0
K06911	5484.24	-0.97	0.37	0
K12297	962.89	-1.79	0.68	0
K07223	1695.47	-1.84	0.70	0
K03478	580.53	-1.92	0.73	0
K08224	1358.71	-1.72	0.66	0
K09908	714.14	-2.22	0.86	0
K09906	714.14	-2.22	0.86	0
K08992	714.14	-2.22	0.86	0
K06205	714.14	-2.22	0.86	0
K07153	714.14	-2.22	0.86	0
K05365	1428.03	-2.22	0.85	0
K05809	714.14	-2.22	0.86	0
K05803	714.14	-2.22	0.86	0
K03683	714.14	-2.22	0.86	0
K03748	727.48	-2.21	0.85	0
K00631	714.14	-2.22	0.86	0
K07724	677.07	-2.35	0.90	0
K03548	714.14	-2.22	0.86	0
K11991	714.14	-2.22	0.86	0
K07048	976.00	-1.36	0.52	0
K05787	714.14	-2.22	0.86	0
K06149	714.14	-2.22	0.86	0
K07235	714.14	-2.22	0.86	0
K07237	714.14	-2.22	0.86	0
K07236	714.14	-2.22	0.86	0

K02504	714.14	-2.22	0.86	0
K02507	749.47	-1.99	0.76	0
K07062	1187.39	-1.56	0.60	0
K03804	714.14	-2.22	0.86	0
K09891	714.14	-2.22	0.86	0
K09899	714.14	-2.22	0.86	0
K07648	714.14	-2.22	0.86	0
K05873	474.06	-2.65	1.02	0
K03457	959.87	-1.49	0.57	0
K02851	714.14	-2.22	0.86	0
K00374	812.74	-1.44	0.55	0
K02682	714.14	-2.22	0.86	0
K08306	919.25	-1.84	0.71	0
K04774	714.14	-2.22	0.86	0
K02344	714.14	-2.22	0.86	0
K03633	714.14	-2.22	0.86	0
K09998	714.14	-2.22	0.86	0
K09999	714.14	-2.22	0.86	0
K03573	714.14	-2.22	0.86	0
K07735	1625.80	-1.55	0.59	0
K07751	714.14	-2.22	0.86	0
K04088	1646.91	-0.93	0.36	0
K07773	714.14	-2.22	0.86	0
K04691	714.14	-2.22	0.86	0
K10000	714.14	-2.22	0.86	0
K00247	714.14	-2.22	0.86	0
K02575	1329.43	-1.46	0.56	0
K03607	714.14	-2.22	0.86	0
K11066	686.68	-2.50	0.95	0
K05838	937.27	-1.89	0.73	0
K08223	620.81	-2.00	0.77	0
K00799	2655.13	-1.89	0.73	0
K03568	1428.47	-1.50	0.58	0
K14415	603.27	-1.89	0.73	0
K03592	1419.99	-1.52	0.59	0
K03646	787.58	-1.83	0.71	0
K00813	949.54	-1.96	0.76	0
K03230	666.03	-2.43	0.95	0
K03219	666.03	-2.43	0.95	0
K02444	1802.72	-0.78	0.30	0
K03580	934.53	-1.88	0.74	0
K03087	750.06	-2.31	0.90	0
K11607	706.75	-2.22	0.86	0
K11606	706.75	-2.22	0.86	0
K11605	706.75	-2.22	0.86	0
K11604	706.75	-2.22	0.86	0

K12150	664.67	-2.44	0.95	0
K02298	666.03	-2.43	0.95	0
K02299	666.03	-2.43	0.95	0
K02297	666.03	-2.43	0.95	0
K01112	664.67	-2.44	0.95	0
K03184	706.75	-2.22	0.86	0
K03181	708.00	-2.21	0.86	0
K02300	666.03	-2.43	0.95	0
K03651	934.53	-1.88	0.74	0
K03228	666.03	-2.43	0.95	0
K03229	666.03	-2.43	0.95	0
K03226	666.03	-2.43	0.95	0
K03227	666.03	-2.43	0.95	0
K03224	666.03	-2.43	0.95	0
K13735	664.67	-2.44	0.95	0
K07121	934.52	-1.91	0.74	0
K04770	933.33	-1.91	0.74	0
K03690	708.00	-2.21	0.86	0
K03835	828.84	-1.88	0.73	0
K02819	1150.70	-1.09	0.42	0
K03490	701.88	-1.52	0.59	0
K11743	1477.90	-1.32	0.52	0
K07687	667.21	-2.42	0.95	0
K02858	782.65	-1.84	0.72	0
K03414	565.24	-2.43	0.95	0
K01716	930.42	-1.92	0.75	0
K03317	904.66	-1.63	0.64	0
K08357	569.08	-2.30	0.90	0
K00989	2380.12	-0.84	0.33	0
K03320	2491.62	-0.95	0.37	0
K05590	927.68	-1.92	0.76	0
K09902	927.68	-1.92	0.76	0
K03645	927.68	-1.92	0.76	0
K07400	927.68	-1.92	0.76	0
K02062	929.42	-1.91	0.75	0
K02679	927.68	-1.92	0.76	0
K08312	927.68	-1.92	0.76	0
K03586	929.03	-1.91	0.76	0
K03764	927.68	-1.92	0.76	0
K08162	559.45	-2.00	0.79	0
K03528	927.68	-1.92	0.76	0
K02521	718.33	-2.17	0.86	0
K02560	927.68	-1.92	0.76	0
K07662	927.68	-1.92	0.76	0
K09897	927.68	-1.92	0.76	0
K09893	927.68	-1.92	0.76	0

K09892	927.68	-1.92	0.76	0
K09911	927.68	-1.92	0.76	0
K03674	927.68	-1.92	0.76	0
K05851	927.68	-1.92	0.76	0
K06957	927.68	-1.92	0.76	0
K02680	927.68	-1.92	0.76	0
K07122	929.03	-1.91	0.76	0
K03632	797.96	-2.14	0.84	0
K03597	929.03	-1.91	0.76	0
K03591	927.68	-1.92	0.76	0
K01713	43.61	-1.75	0.69	0
K07479	927.68	-1.92	0.76	0
K01608	690.58	-1.56	0.61	0
K07184	927.68	-1.92	0.76	0
K02839	559.45	-2.00	0.79	0
K06145	930.23	-1.91	0.75	0
K06899	929.35	-1.91	0.76	0
K09997	733.60	-2.15	0.85	0
K02199	1004.34	-1.79	0.71	0
K01218	1516.65	1.30	0.52	0
K09136	1041.99	-1.55	0.62	0
K03598	1013.12	-1.88	0.75	0
K06905	1843.96	-1.10	0.44	0
K02779	1227.07	-1.16	0.47	0
K02505	737.73	-2.04	0.82	0
K11179	767.13	-1.86	0.75	0
K11905	475.32	-2.69	1.08	0
K03658	562.44	-1.96	0.79	0
K00325	786.81	-1.80	0.73	0
K00230	1060.14	-1.43	0.58	0
K03834	743.74	-2.11	0.85	0
K11895	476.52	-2.67	1.08	0
K11892	476.52	-2.67	1.08	0
K11891	476.52	-2.67	1.08	0
K01093	737.85	-1.96	0.79	0
K11894	474.48	-2.68	1.09	0
K11910	474.48	-2.68	1.09	0
K01816	847.43	-1.74	0.71	0
K11737	474.48	-2.68	1.09	0
K07266	935.74	-1.35	0.55	0
K07265	935.74	-1.35	0.55	0
K06995	468.26	-2.68	1.09	0
K07071	877.41	-1.75	0.71	0
K12137	68.68	1.52	0.62	0
K13542	7329.45	0.42	0.17	0
K07789	475.68	-2.66	1.09	0

K07788	475.68	-2.66	1.09	0
K11214	478.08	1.02	0.42	0
K03089	1566.89	-1.34	0.55	0
K02509	619.64	-1.66	0.68	0
K11747	957.45	-1.85	0.76	0
K09800	1538.24	-1.35	0.56	0
K02821	1579.14	-1.17	0.48	0
K11893	483.30	-2.57	1.07	0
K02022	1365.53	-1.34	0.56	0
K08358	652.98	-2.18	0.91	0
K05966	758.99	-1.65	0.69	0
K08304	1496.37	-1.41	0.59	0

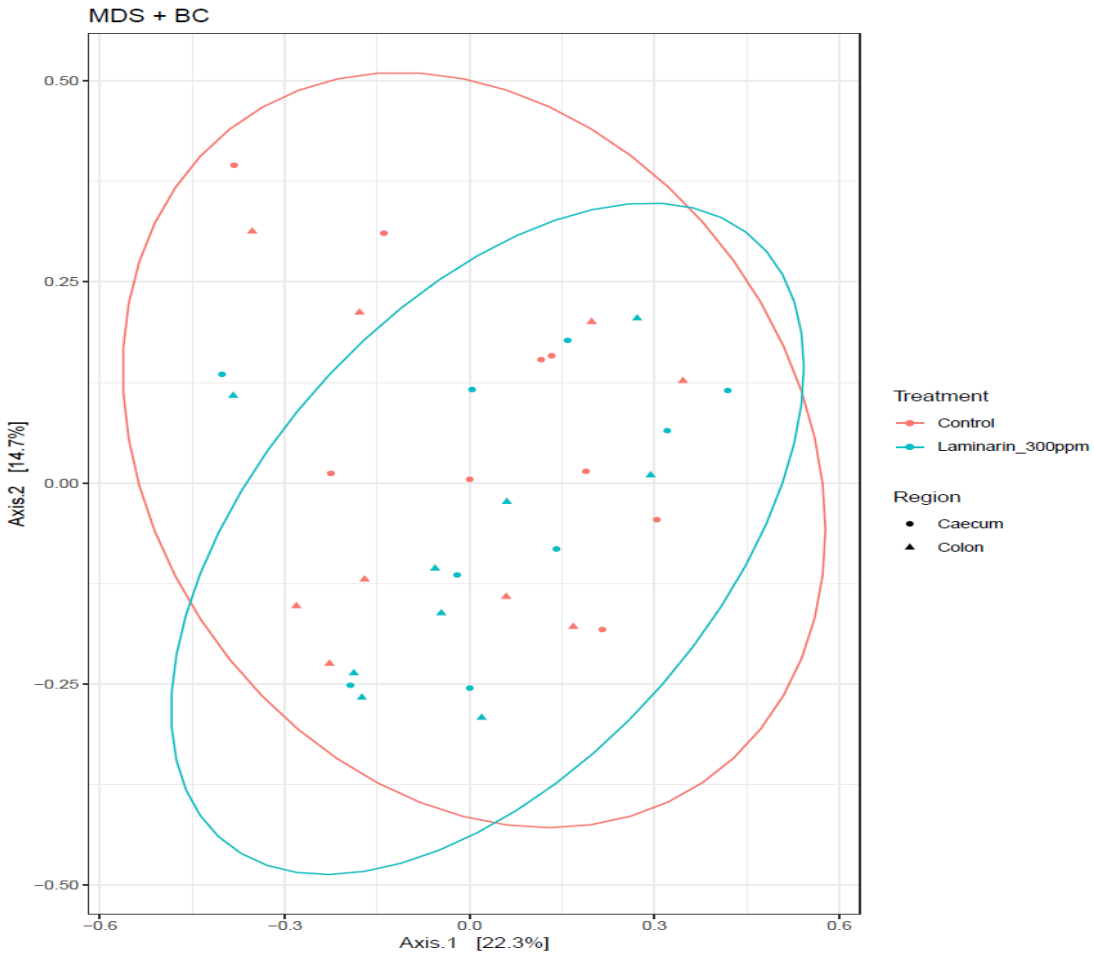

Figure S1: Beta-diversity analysis using multiple dimensional scaling and bray-curtis distance matrix

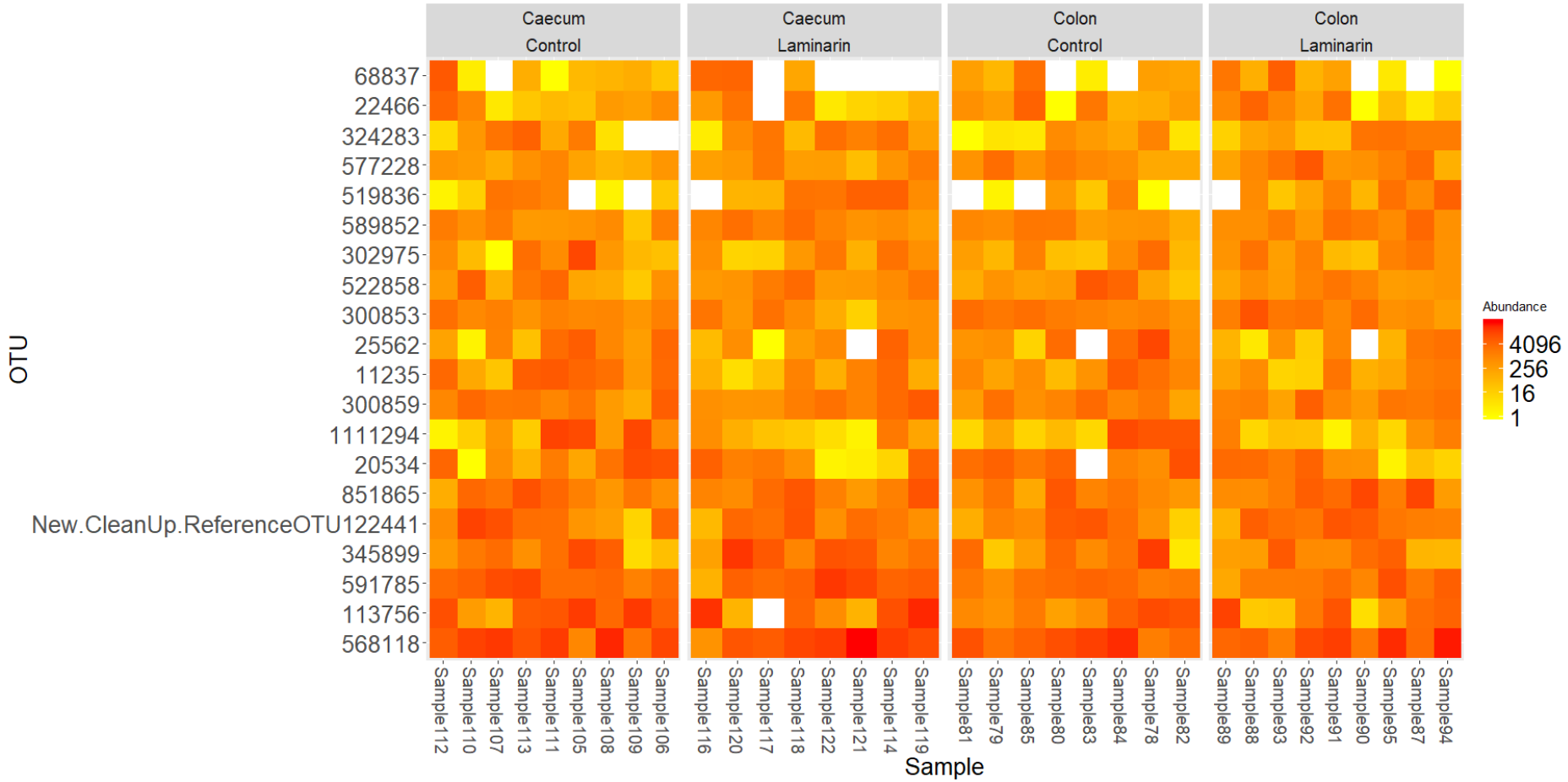

Figure S2: Heatmap showing the top 20 most abundant OTUs

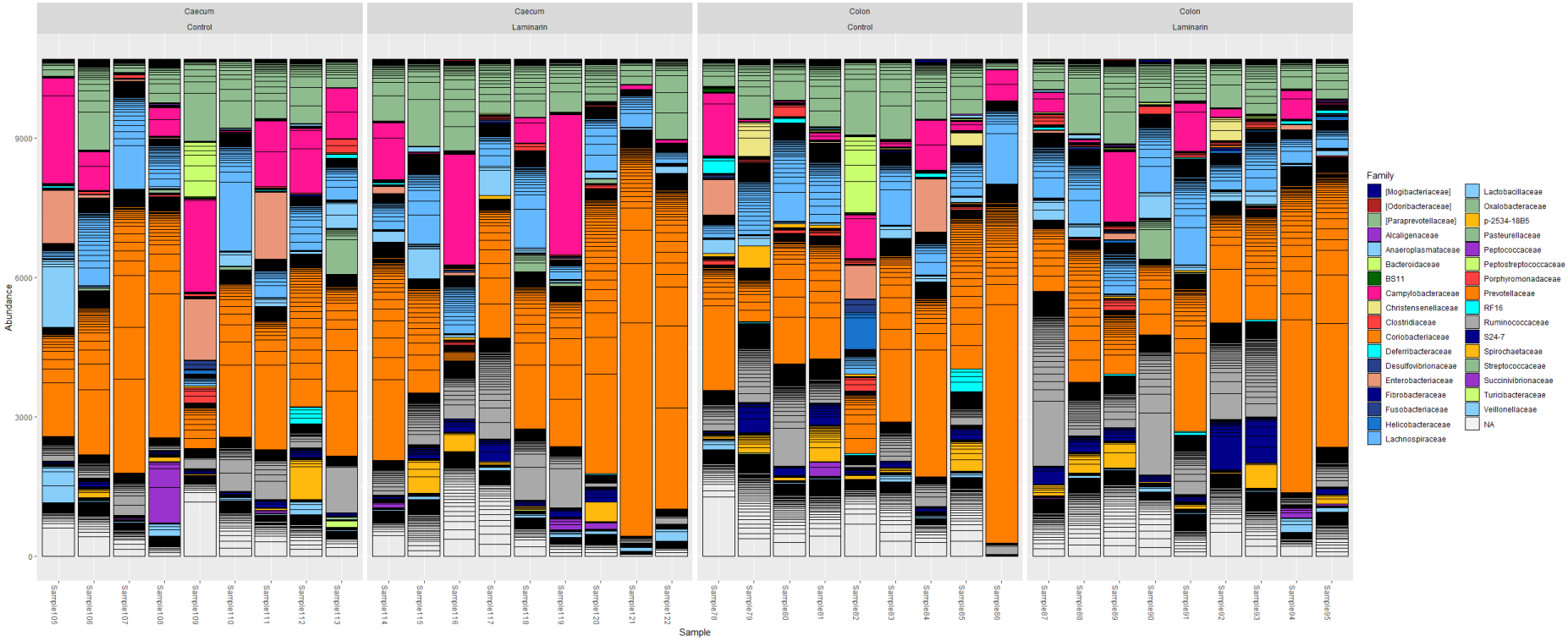

Figure S3: Taxonomic distribution of individual samples at family level
